# A role for the mitotic proteins Bub3 and BuGZ in transcriptional regulation of *catalase-3* expression

**DOI:** 10.1371/journal.pgen.1010254

**Published:** 2022-06-06

**Authors:** Yike Zhou, Shuangjie Shen, Chengcheng Du, Ying Wang, Yi Liu, Qun He

**Affiliations:** 1 State Key Laboratory of Agrobiotechnology and MOA Key Laboratory of Soil Microbiology, College of Biological Sciences, China Agricultural University, Beijing, China; 2 Department of Physiology, The University of Texas Southwestern Medical Center, Dallas, Texas, United States of America; University of Georgia, UNITED STATES

## Abstract

The spindle assembly checkpoint factors Bub3 and BuGZ play critical roles in mitotic process, but little is known about their roles in other cellular processes in eukaryotes. In aerobic organisms, transcriptional regulation of *catalase* genes in response to developmental or environmental stimuli is necessary for redox homeostasis. Here, we demonstrate that Bub3 and BuGZ negatively regulate *cat-3* transcription in the model filamentous fungus *Neurospora crassa*. The absence of Bub3 caused a significant decrease in BuGZ protein levels. Our data indicate that BuGZ and Bub3 interact directly via the GLEBS domain of BuGZ. Despite loss of the interaction, the amount of BuGZ mutant protein negatively correlated with the *cat-3* expression level, indicating that BuGZ amount rather than Bub3-BuGZ interaction determines *cat-3* transcription level. Further experiments demonstrated that BuGZ binds directly to the *cat-3* gene and responses to *cat-3* overexpression induced by oxidative stresses. However, the zinc finger domains of BuGZ have no effects on DNA binding, although mutations of these highly conserved domains lead to loss of *cat-3* repression. The deposition of BuGZ along *cat-3* chromatin hindered the recruitment of transcription activators GCN4/CPC1 and NC2 complex, thereby preventing the assembly of the transcriptional machinery. Taken together, our results establish a mechanism for how mitotic proteins Bub3 and BuGZ functions in transcriptional regulation in a eukaryotic organism.

## Author summary

The spindle assembly checkpoint is a key node in cell cycle during mitosis or meiosis which is responsible for proper chromosome segregation, and Bub3 along with its recently identified chaperon BuGZ are typical components in this biological process. Here, we discover a novel function of this couple of proteins in transcriptional regulation of *catalase* genes through extensive genetic screening in filamentous fungus *Neurospora crassa*. To our surprise, the regulatory relationship between Bub3 and BuGZ in fungi is quite different from that in higher eukaryotes, which may be attributed to the discrepancy that occur over long evolutionary stages. Moreover, the Bub3-BuGZ interaction which is essential for the checkpoint function shows insignificance in gene regulation. In contrast, the amount of BuGZ proved to be the key factor. In addition to DNA binding capacity, BuGZ is able to respond to oxidative stress and specifically inhibit gene overexpression. Besides, the conserved zinc finger domains of BuGZ also play vital roles in transcriptional repression beyond its classical DNA binding function. Our results not only expand the mechanism of *catalase* transcriptional regulation for maintaining redox homeostasis, but also indicate the multifunctionality of traditional mitotic proteins in life activity.

## Introduction

Reactive oxygen species (ROS) are byproducts of oxygen consumption and cellular metabolism in aerobic organisms [1,2]. ROS are a heterogeneous group of highly reactive ions and molecules derived from oxygen, including superoxide anion, hydrogen peroxide (H_2_O_2_), hydroxyl radicals, and singlet oxygen, which have inherent chemical properties that confer reactivity to different biological targets [3,4]. ROS can be deleterious or beneficial [5,6]. Excess toxic ROS can cause oxidation damage to macromolecules such as DNA, lipids, and proteins and underlie a wide spectrum of human disorders [7–10]. It is crucial that excess ROS be removed to maintain redox homeostasis [11,12]. Detoxification of ROS is achieved by enzymatic antioxidants such as superoxide dismutase, catalase, peroxiredoxin, glutathione peroxidase, and glutathione reductase [13,14]. Catalases are necessary for conversion of H_2_O_2_ to innocuous dioxygen and water and are highly conserved and precisely regulated at multiple levels in aerobionts to achieve redox homeostasis [15].

The filamentous fungus *Neurospora crassa* has three monofunctional catalases, CAT-1, CAT-3 and CAT-4, and one catalase peroxidase, CAT-2 [16]. CAT-1 and CAT-3 are large-subunit catalases that are responsible for most catalase activities. The activities of CAT-1 and CAT-3 are differentially regulated during the asexual life cycle [16,17]. CAT-1 is associated with non-growing cells and accumulates in asexual spores (conidia), whereas CAT-3 activity increases during exponential growth and is induced under different stress conditions [16–20]. CAT-3 is present in growing mycelia, where it mediates resistance to oxidative stress; its function cannot be replaced by other catalases [20]. Our previous studies have identified several transcriptional regulators of *cat-3* expression. For example, GCN4/CPC1, a multifunctional regulator, coordinates with histone acetyltransferase GCN5 to activate *cat-3* expression under oxidative stress [21], and NC2 complex activates *cat-3* transcription by promoting INO80C-mediated H2A.Z removal on chromatin [22]. However, the regulatory mechanism of *cat-3* transcriptional repression process is almost unknown.

The spindle assembly checkpoint (SAC), also known as the mitotic checkpoint, is a cell-cycle checkpoint that maintains genome stability by preventing the separation of the duplicated chromosomes until each chromosome is properly attached to the spindle via its kinetochore [23–27]. The molecular components of this pathway were first identified in *S*. *cerevisiae* and subsequently in higher eukaryotic organisms. The main components of the SAC are Bub and Mad family members [28–31]. The Bub proteins are components of the mitotic checkpoint complex, which inhibits the activity of anaphase-promoting complex/cyclosome (APC/C) and establishes correct kinetochore-microtubule attachments [32]. As a pioneering factor that recruits checkpoint proteins to the kinetochore, Bub3 interacts with Bub1 or BubR1 through their highly conserved GLE-2-binding sequence (GLEBS) domains; the interaction with Bub3 is critical for their loading on the kinetochore [26,33]. Recently, BuGZ (also known as ZNF207), a novel GLEBS-containing protein that interacts with Bub3 was identified [34,35]. BuGZ is required for kinetochore loading of Bub3 and is necessary for proper chromosome alignment and mitotic progression [34–36]. A phase transition of BuGZ promotes microtubule polymerization and spindle apparatus assembly, which is critical for activation of the mitotic kinase AurA [37,38]. In addition, Bub3 and BuGZ are also implicated in the occurrence of cancer [39–42].

Bub3 and BuGZ are also known to function in other biological processes together or separately. For example, in the interphase nucleus, BuGZ and Bub3 interact with the splicing machinery and are required for pre-mRNA splicing and inhibition of R-loop-mediated DNA damage to human cells [43,44]. Moreover, Bub3 is involved in the process of telomere DNA replication, DNA methylation modification, and gene transcription inhibition [45–47]. An increasing body of evidence suggests that BuGZ acts as a transcription factor to regulate gene expression in different organisms [48–50]. The identities of direct downstream targets of BuGZ in human embryonic stem cells suggest that BuGZ functions in cell-cycle regulation, ectoderm development, and stem cell signaling pathways [49]. Nonetheless, how BuGZ acts to regulate gene expression is still unclear.

Here, we investigated the functions of Bub3 and BuGZ in *N*. *crassa*. We found that Bub3 represses *cat-3* transcription by maintaining the stability of BuGZ. BuGZ specifically binds to the *cat-3* gene and shows increased recruitment in response to overexpressed *cat-3* stimulated by oxidative stresses. The conserved N-terminal zinc finger domains of BuGZ are crucial for transcriptional repression but useless for DNA binding. Deletion of BuGZ led to increased recruitment of transcription activators GCN4/CPC1 and NC2 complex to regulatory regions of the *cat-3* gene. Taken together, our results reveal how mitotic proteins Bub3 and BuGZ function in the regulation of intracellular redox homeostasis by controlling *cat-3* expression. Our work also establishes the mechanism for the role of Bub3 and BuGZ in transcriptional regulation.

## Results

### *bub3* mutants resist H_2_O_2_-induced ROS stress and have elevated *cat-3* expression

**Fig 1:**
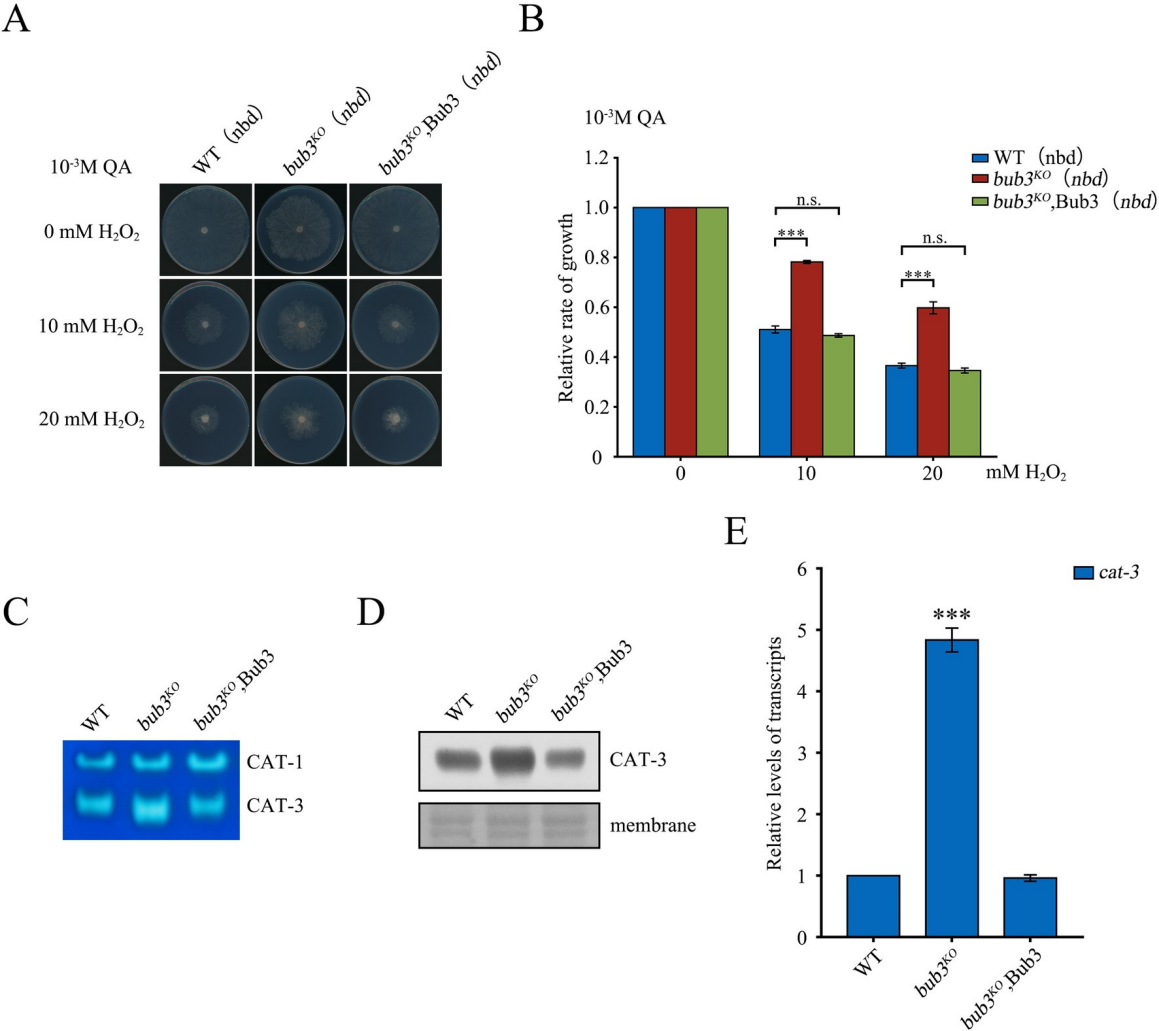
Bub3 functions to suppress *cat-3* gene expression. (A) Mycelial growth of WT, *bub3*^*KO*^, and *bub3*^*KO*^,Bub3 (*nbd*) strains on plates with addition of 1x10^-3^ M QA and 0, 10, or 20 mM H_2_O_2_. (B) Quantitation of growth relative to WT of *bub3*^*KO*^ and *bub3*^*KO*^,Bub3 (*nbd*) strains under conditions described in panel A. (C) In-gel catalase activity assay of protein extracts from WT, *bub3*^*KO*^, and *bub3*^*KO*^,Bub3 strains. (D) The level of CAT-3 protein in WT, *bub3*^*KO*^, and *bub3*^*KO*^,Bub3 strains determined by western blot analyses. The membranes stained by Coomassie blue served as the loading control. (E) Levels of *cat-3* mRNA in *bub3*^*KO*^ and *bub3*^*KO*^,Bub3 strains relative to that in the WT strain as determined by RT-qPCR analyses.Cultures were inoculated at the center of petri dishes (9 cm in diameter) and grown at 25°C under constant light. Error bars indicate SD (n = 3). Significance was evaluated by two-tailed *t*-test. *P < 0.05, **P < 0.01, and ***P<0.001.

To identify the regulatory factors involved in coping with oxidative stress, we examined the H_2_O_2_ sensitivity of available single gene deletion mutants of *N*. *crassa*. The strain with a *bub3* deletion (*bub3*^*KO*^, NCU09744) exhibited significant enhanced resistance to H_2_O_2_ compared with the wild-type (WT) strain (S1A and S1B Fig). Amino acid sequence alignment showed that the Bub3 is highly conserved among eukaryotes (S2A Fig). To confirm the function of Bub3 in H_2_O_2_ resistance, a plasmid carrying the sequence encoding Myc-Bub3 driven by a promoter inducible by quinic acid (QA) was introduced into the *bub3*^*KO*^ strain. The ectopic expression of Myc-Bub3 and addition of QA restored wild-type levels of growth and the sensitivity to H_2_O_2_ (Fig 1A and 1B), indicating that the observed H_2_O_2_-resistant phenotype of the *bub3*^*KO*^ strain was due to loss of Bub3. It should be noted that the rescue phenotype was only seen when the *bub3*^*KO*^ was in the *nonband* (*nbd*) background which has a wild-type *ras-1* gene [51]. Thus, subsequent experiments related to *bub3*^*KO*^ transformants were carried out in this genetic background.

Since catalases are responsible for catalyzing the breakdown of H_2_O_2_ into water and oxygen in *N*. *crassa*, these results suggested that Bub3 regulates catalase expression in response to H_2_O_2_-induced oxidative stress. Bub3 is a component of kinetochore-bound SAC complex [28–31], but little is known about its transcriptional function. To determine whether Bub3 directly affects the activity of catalases, we used a zymogram in-gel assay to evaluate catalase activity in WT and *bub3*^*KO*^ strains. In the mutant, there was a slightly enhancement of activity of CAT-1 and ~ 5-fold increase of CAT-3 activity compared to their activities in the WT strain (S1C Fig), suggesting that the H_2_O_2_-resistant phenotype observed in mutants was probably due to dramatically elevated CAT-3 activity. Western blot analyses revealed that the up-regulation of CAT-3 activity in mutants resulted from increased CAT-3 protein levels (S1D Fig), indicating that Bub3 is required for repression of CAT-3 expression. RT-qPCR analyses also showed a significant increase of *cat-3* mRNA in the *bub3*^*KO*^ strain compared to that in WT strain (S1E Fig). Moreover, ectopic expression of Myc-Bub3 reduced CAT-3 protein and *cat-3* mRNA expression in the *bub3*^*KO*^ strain to the WT levels (Fig 1C–1E). Taken together, these results indicate that Bub3 is a negative regulator of *cat-3* expression.

### BuGZ functions as a negative regulator of *cat-3* gene expression

**Fig 2:**
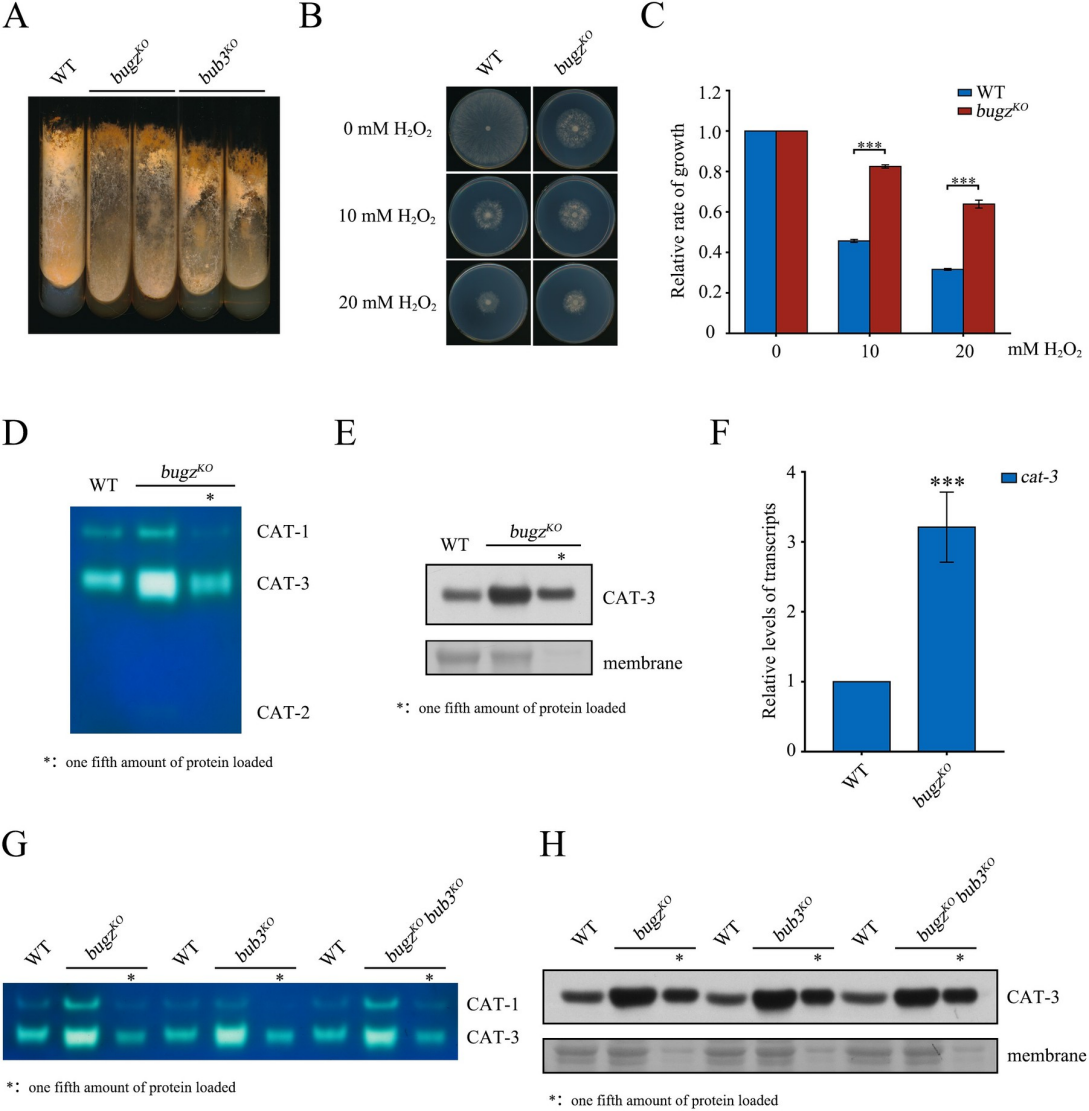
BuGZ and Bub3 function in the same pathway to repress *cat-3* expression. (A) Aerial hyphae and conidia of WT, *bugz*^*KO*^, and *bub3*^*KO*^ strains on slants. Cultures were grown at 25°C under constant light after 1 day at 30°C. (B) Mycelial growth of WT and *bugz*^*KO*^ strains on plates with 0, 10, or 20 mM H_2_O_2_. (C) Quantitation of growth of *bugz*^*KO*^ strain relative to WT under conditions described in panel B. (D) In-gel catalase activity assays of protein extracts from WT and *bugz*^*KO*^ strains. (E) Western blot analysis of CAT-3 protein in WT and *bugz*^*KO*^ strains. The membranes stained by Coomassie blue served as the loading control. (F) Quantification of *cat-3* mRNA in *bugz*^*KO*^ strain relative to WT. (G) In-gel catalase activity assays of protein extracts from WT, *bugz*^*KO*^, *bub3*^*KO*^, and *bugz*^*KO*^*bub3*^*KO*^ strains. (H) Western blot analysis of CAT-3 protein in WT, *bugz*^*KO*^, *bub3*^*KO*^, and *bugz*^*KO*^*bub3*^*KO*^ strains. The membranes stained by Coomassie blue served as the loading control. Error bars indicate SD (n = 3). Significance was evaluated by two-tailed *t*-test. *P < 0.05, **P < 0.01, and ***P<0.001.

In higher eukaryotes, the interaction between Bub3 and its chaperone BuGZ contributes to the stabilization and kinetochore loading of Bub3, to achieve proper chromosome alignment and mitotic progression during cell division [34–36, 39, 52, 53]. Although BuGZ homolog is conserved in filamentous fungi (S2B Fig), they are not found in budding and fission yeasts [35]. In *N*. *crassa*, deletion of *bugz* (NCU06145, previously known as *all development altered-21*) [54] resulted in abnormalities in mycelial growth, hyphal formation, and conidial development that were more severe than those of the *bub3*^*KO*^ strain (Fig 2A). Like the *bub3*^*KO*^ strain, the *bugz*^*KO*^ strain had an H_2_O_2_-resistant phenotype (Fig 2B and 2C). Moreover, the activity and the levels of *cat-3* mRNA and protein were also significantly increased in the *bugz*^*KO*^ strain (Fig 2D–2F). These data indicate that BuGZ, like Bub3, is required for suppression of *cat-3* transcription. Considering that there are other enzymes important for H_2_O_2_ detoxification such as the peroxiredoxins, which may be regulated by Bub3 or BuGZ and involved in H_2_O_2_-resistance, we further created the *bub3*^*KO*^ *cat-3*^*KO*^ and *bugz*^*KO*^ *cat-3*^*KO*^ double mutants. It is obvious that they both exhibit a H_2_O_2_-sensitive phenotype similar to that of *cat-3*^*KO*^ strain (S3 Fig), confirming that the H_2_O_2_-resistant phenotype in *bub3*^*KO*^ or *bugz*^*KO*^ mutants was predominantly due to dramatically elevated CAT-3 activity.

The function of BuGZ in H_2_O_2_ resistance through regulation of *cat-3* transcription was confirmed genetically. Ectopic expression of Myc-BuGZ in the *bugz*^*KO*^ strain resulted in WT-level growth and the sensitivity to H_2_O_2_ (S4A and S4B Fig). Ectopic expression of Myc-BuGZ in the *bugz*^*KO*^ strain also efficiently suppressed *cat-3* expression to the WT level (S4C–S4E Fig). To confirm the relationship between BuGZ and Bub3 in regulation of *cat-3* expression, we generated the *bugz*^*KO*^*bub3*^*KO*^ double mutant. The activity and protein levels of CAT-3 in the double mutant were the same within experimental error as in the single mutants (Fig 2G and 2H), which indicates that BuGZ and Bub3 function in the same pathway to regulate *cat-3* expression.

### The level of BuGZ protein, but not the Bub3-BuGZ interaction, is responsible for repressing *cat-3* expression

**Fig 3:**
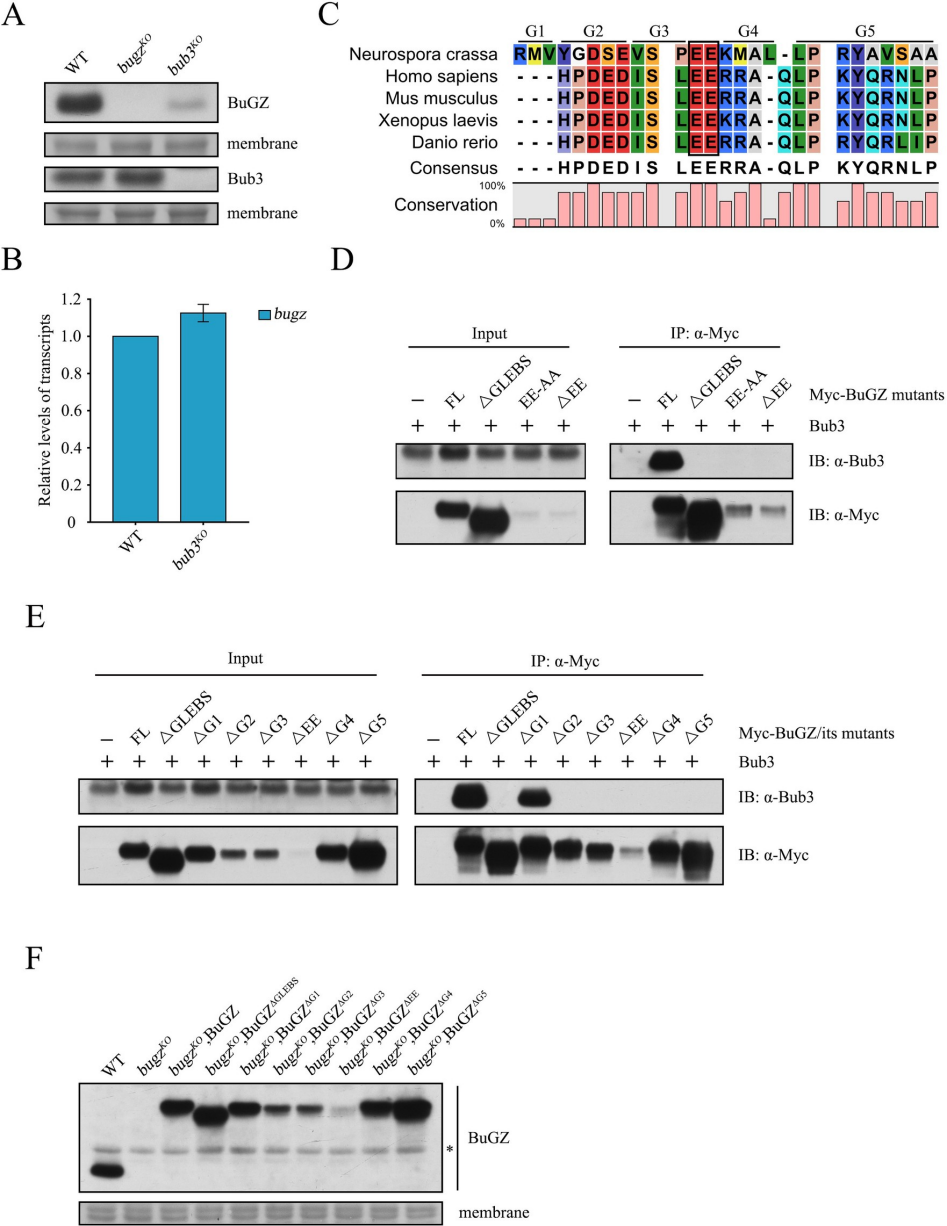
BuGZ stability does not depend on the Bub3-BuGZ interaction. (A) Western blot for BuGZ and Bub3 proteins in WT, *bugz*^*KO*^, and *bub3*^*KO*^ strains. The membranes stained by Coomassie blue served as the loading control. (B) RT-qPCR quantification of *bugz* mRNA in *bub3*^*KO*^ strain relative to WT. (C) Amino acid sequence alignment of the conserved GLEBS domain of BuGZ from *Neurospora crassa*, *Homo sapiens*, *Mus musculus*, *Xenopus laevis*, and *Danio rerio*. The conserved EE residues are indicated by a black box, and G1~G5 segments are marked. (D) Co-immunoprecipitation assays analyzed by western blot to evaluate the interaction between Myc-BuGZ, Myc-BuGZ^ΔGLEBS^, Myc-BuGZ^EE-AA^, or Myc-BuGZ^ΔEE^ and endogenous Bub3. (E) Co-immunoprecipitation assays analyzed by western blot to evaluate the interaction between Myc-BuGZ, Myc-BuGZ^ΔGLEBS^, Myc-BuGZ^ΔG1~ΔG5^, or Myc-BuGZ^ΔEE^ and endogenous Bub3. (F) Western blot analyses of the levels of BuGZ protein in WT, *bugz*^*KO*^, *bugz*^*KO*^,BuGZ, *bugz*^*KO*^,BuGZ^ΔGLEBS^, *bugz*^*KO*^,BuGZ^ΔG1~ΔG5^, and *bugz*^*KO*^,BuGZ^ΔEE^ strains. The membranes stained by Coomassie blue served as the loading control. A non-specific protein band is marked by an asterisk. Error bars indicate SD (n = 3). Significance was evaluated by two-tailed *t*-test. *P < 0.05, **P < 0.01, and ***P<0.001.

To determine how BuGZ and Bub3 coordinate to regulate the expression of *cat-3*, we generated polyclonal Bub3- and BuGZ-specific antibodies, which specifically recognized the endogenous protein based on predicted molecular weight in the WT strain but not in the knock-out strains (S5 Fig). To our surprise, the western blot results showed that although the level of Bub3 was slightly increased in the *bugz*^*KO*^ strains, the lack of Bub3 led to considerable reduction in levels of BuGZ compared to that present in the WT strain (Fig 3A). This is quite different from the situation in higher organisms, in which BuGZ is required for Bub3 stability [34,35]. Since the deletion of *bub3* did not cause the down-regulation of *bugz* expression (Fig 3B), these results suggest that Bub3 stabilizes BuGZ protein at post-transcriptional level in *N*. *crassa*.

Previous studies have shown that the GLEBS domain of BuGZ forms a series of salt bridges with the WD40 domains of Bub3; two conserved glutamate residues in the GLEBS domain are key to the interaction with Bub3 [34,55]. The GLEBS domain of BuGZ in *N*. *crassa* shares high homology with the same region in higher eukaryotes with the glutamate residues conserved (Fig 3C). To test the importance of these glutamates to the interaction with Bub3, we generated *qa-2* promoter driven Myc-tagged BuGZ constructs with the GLEBS domain deleted or the conserved glutamates mutated and introduced into the *bugz*^*KO*^ strain. A co-immunoprecipitation assay verified that all mutant BuGZ proteins had lost the ability to interact with Bub3 (Fig 3D). In cells with an EE to AA mutation or with an EE deletion, levels of the Myc-tagged mutant BuGZ was considerably reduced compared to the Myc-tagged wild-type BuGZ; however, deletion of the entire GLEBS domain resulted in higher levels of BuGZ (Fig 3D). We next generated a series of strains with deletion of different segments inside the GLEBS domain of BuGZ (Fig 3C). The interaction between Myc-BuGZ^ΔG1^ and Bub3 was weakened compared to that between the Myc-tagged wild-type BuGZ and Bub3, while the lack of all other segments resulted in complete loss of interaction between the two proteins (Fig 3E). Except for the high BuGZ level in the strain lacking the entire GLEBS domain, the amount of BuGZ protein varied among the strains with deficiency of Bub3-BuGZ interaction (Fig 3E and 3F). These data suggest that the interaction between BuGZ and Bub3 is not a determinant in maintaining BuGZ protein stability.

**Fig 4:**
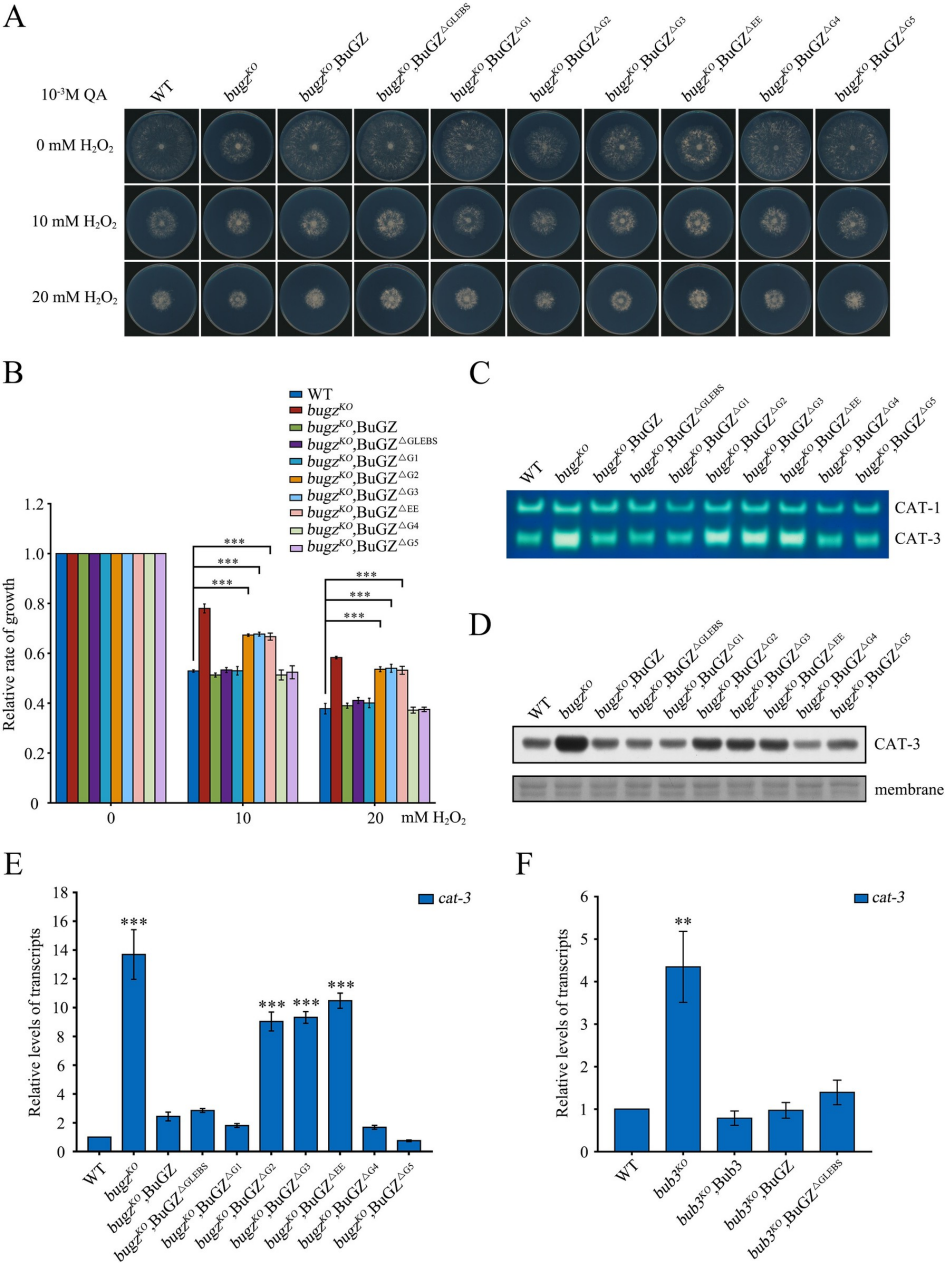
BuGZ protein level determines extent of *cat-3* transcription inhibition. (A) Mycelial growth of WT, *bugz*^*KO*^, *bugz*^*KO*^,BuGZ, *bugz*^*KO*^,BuGZ^ΔGLEBS^, *bugz*^*KO*^,BuGZ^ΔG1~ΔG5^, and *bugz*^*KO*^,BuGZ^ΔEE^ strains on plates with 0, 10, or 20 mM H_2_O_2_. (B) Quantitation of growth of strains described in panel A relative to WT. (C) In-gel analyses of catalases activities in extracts from WT, *bugz*^*KO*^, *bugz*^*KO*^,BuGZ, *bugz*^*KO*^,BuGZ^ΔGLEBS^, *bugz*^*KO*^,BuGZ^ΔG1~ΔG5^, and *bugz*^*KO*^,BuGZ^ΔEE^ strains. (D) Western blot analysis of CAT-3 protein in WT, *bugz*^*KO*^, *bugz*^*KO*^,BuGZ, *bugz*^*KO*^,BuGZ^ΔGLEBS^, *bugz*^*KO*^,BuGZ^ΔG1~ΔG5^, and *bugz*^*KO*^,BuGZ^ΔEE^ strains. The membranes stained by Coomassie blue served as the loading control. (E) RT-qPCR analyses of *cat-3* mRNA in *bugz*^*KO*^, *bugz*^*KO*^,BuGZ, *bugz*^*KO*^,BuGZ^ΔGLEBS^, *bugz*^*KO*^,BuGZ^ΔG1~ΔG5^, and *bugz*^*KO*^,BuGZ^ΔEE^ strains relative to levels in the WT strain. (F) RT-qPCR analyses of *cat-3* mRNA in *bub3*^*KO*^, *bub3*^*KO*^,Bub3, *bub3*^*KO*^,BuGZ, and *bub3*^*KO*^,BuGZ^ΔGLEBS^ strains relative to levels in the WT strain. Error bars indicate SD (n = 3). Significance was evaluated by two-tailed *t*-test. *P < 0.05, **P < 0.01, and ***P<0.001.

To explore the effects of the Bub3-BuGZ interaction and BuGZ protein stability on regulation of *cat-3* expression, we evaluated the H_2_O_2_ sensitivity and *cat-3* expression in the *bugz*^*KO*^ strains that express Myc-tagged BuGZ with various deletions in the GLEBS domain. Phenotypic examination and molecular data showed that the mutants with reduced BuGZ protein levels (BuGZ^ΔG2^, BuGZ^ΔG3^, BuGZ^ΔEE^) had significantly enhanced resistance to H_2_O_2_ and displayed elevated *cat-3* expression compared to the WT strain. In contrast, strains with unchanged (BuGZ^ΔG1^, BuGZ^ΔG4^) or up-regulated (BuGZ^ΔGLEBS^, BuGZ^ΔG5^) BuGZ levels had H_2_O_2_ sensitivity and *cat-3* expression comparable to the WT strain (Fig 4A–4E). Importantly, ectopic expression of Myc-BuGZ or of Myc-BuGZ^ΔGLEBS^ in the *bub3*^*KO*^ strain resulted in WT levels of *cat-3* expression (Figs 4F and S6). These results strongly suggest that the level of BuGZ, but not the interaction between BuGZ and Bub3, is important for *cat-3* repression.

### BuGZ binds to the *cat-3* region specifically for presuppression and its association with chromatin is independent of the zinc finger structure

**Fig 5:**
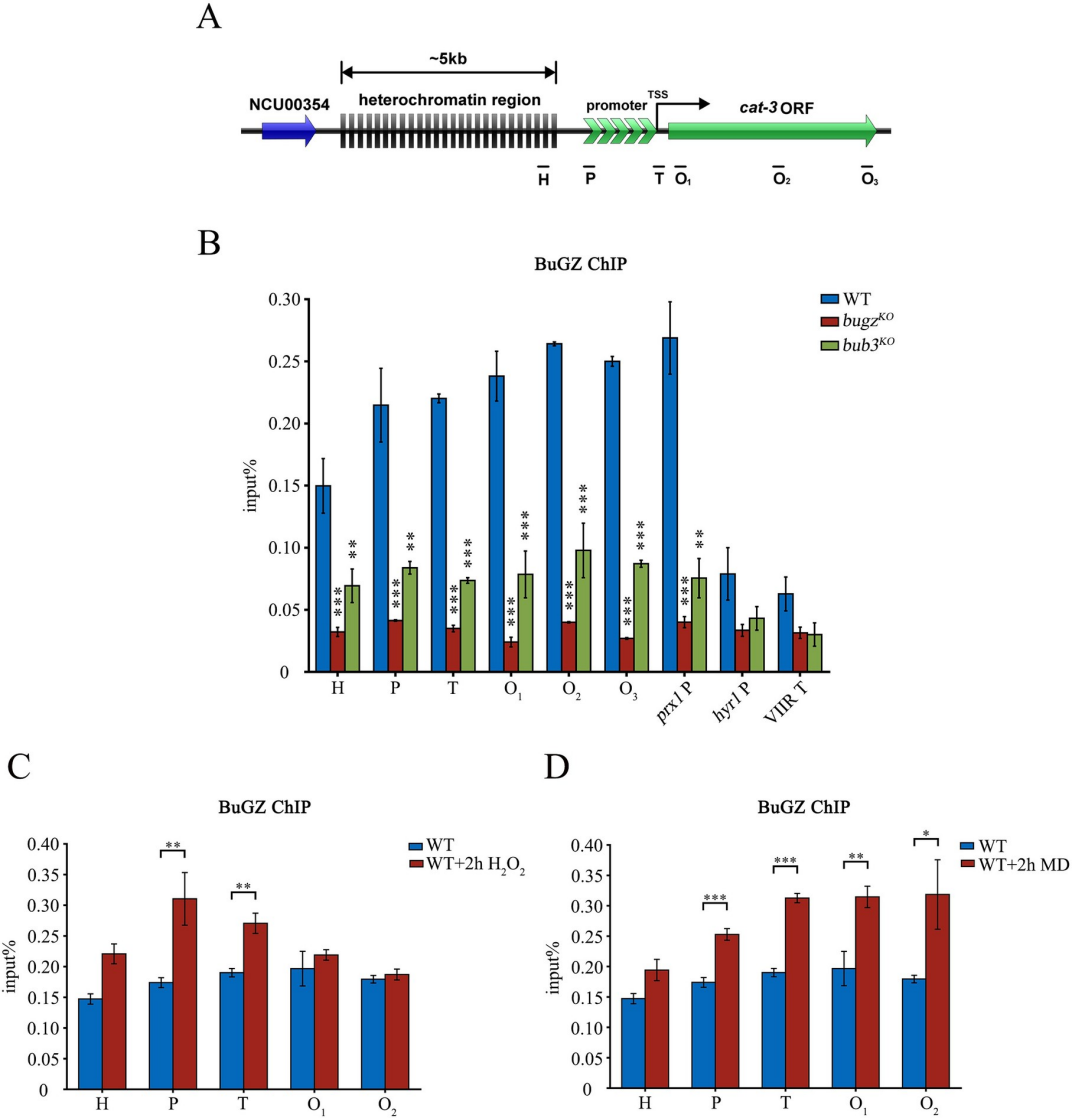
BuGZ binds to the *cat-3* gene region specifically and its recruitment responses to *cat-3* overexpression caused by oxidative stress. (A) Schematic depicting the location of *cat-3* (NCU00355) gene downstream of a 5-kb AT-rich heterochromatin region on linkage group III of *Neurospora* genome. Site of primer binding are indicated under the schematic. Primer H binds in a heterochromatin region; P in the promoter; T near the transcription start site; O_1_, O_2_, and O_3_ in the open reading frame (ORF). (B) Quantification of recruitment of BuGZ to the *cat-3* locus as well as control regions in WT and *bub3*^*KO*^ strains. The *bugz*^*KO*^ strain was used as a negative control. The *prx1* gene promoter region (*prx1* P) was used as a positive binding control for BuGZ, while the *hyr1* gene promoter (*hyr1* P) and a telomeric heterochromatin region on the right arm of chromosome VII (VIIR T) were used as negative control groups. (C, D) ChIP analysis of the binding of BuGZ after (C) H_2_O_2_ or (D) menadione (MD) treatment for 2 hours at *cat-3* locus. Error bars indicate SD (n = 3). Significance was evaluated by two-tailed *t*-test. *P < 0.05, **P < 0.01, and ***P<0.001.

There are two adjacent C2H2-type zinc finger domains in the N-terminal region of BuGZ with very high homology to the same region of the protein in eukaryotes (S2B Fig). This feature suggests that BuGZ homologs may have the ability to bind DNA. To investigate whether BuGZ regulates *cat-3* transcription by directly binding to the *cat-3* gene, we performed ChIP assays using anti-BuGZ antibody and a set of oligonucleotide primer pairs spanning the *cat-3* locus and its upstream heterochromatin region (Fig 5A). Using the *bugz*^*KO*^ strain as a negative control, we found that BuGZ-specific association with chromatin was enriched extensively at the *cat-3* locus in the WT strain. Moreover, in the absence of Bub3, recruitment of BuGZ to *cat-3* locus remained but was significantly reduced compared with that in the WT strain (Fig 5B), which is consistent with the reduced level of BuGZ in the *bub3*^*KO*^ strain.

In *N*. *crassa*, *hyr1* (NCU09534) is the peroxiredoxin coding gene and *prx1* (NCU06031) is another mitochondrial peroxiredoxin coding gene. RT-qPCR analyses showed that BuGZ could negatively regulated *prx1* gene but has no effect on *hyr1* expression. In contrast, Bub3 negatively regulated *hyr1* but not *prx1* expression (S7A Fig), indicating the difference of downstream targets between BuGZ and Bub3. Based on this, we detected the recruitment of BuGZ on the promoter region of *hyr1* or *prx1* for negative or positive binding controls. As we expected, BuGZ binds to the *prx1* promoter significantly, while hardly associates with the *hyr1* promoter as well as a telomeric heterochromatin region (4235800~4255303) on the right arm of *N*. *crassa* chromosome VII used for another negative control region (Fig 5B). These results proved the specificity of BuGZ binding on chromatin.

Previous studies have shown that oxidative stresses can dramatically induce *cat-3* expression [56]. To further examine whether the binding of BuGZ to *cat-3* responds to oxidative stresses, we treated the WT strain with H_2_O_2_ or menadione (MD) for 2 hours and measured the recruitment of BuGZ to *cat-3* locus. Along with the *cat-3* overexpression under oxidative stimulation (S7B Fig), ChIP data revealed that the binding of BuGZ at *cat-3* promoter and TSS was increased in the samples treated with H_2_O_2_ (Fig 5C), while the binding of BuGZ was generally increased at the whole *cat-3* locus in the sample treated with MD (Fig 5D). However, the MD-induced high expression of *prx1* gene resulted in no elevated binding of BuGZ (S7C and S7D Fig), indicating the functional specificity of BuGZ to stimulus response on *cat-3*. These results suggest that highly expressed *cat-3* may create a chromatin environment which is suitable for elevated binding of BuGZ to achieve its repression function after removal of stimuli. Based on this, the basal level of BuGZ binding under physiological conditions is likely to perform a presuppression role for keeping proper *cat-3* expression and redox homeostasis.

**Fig 6:**
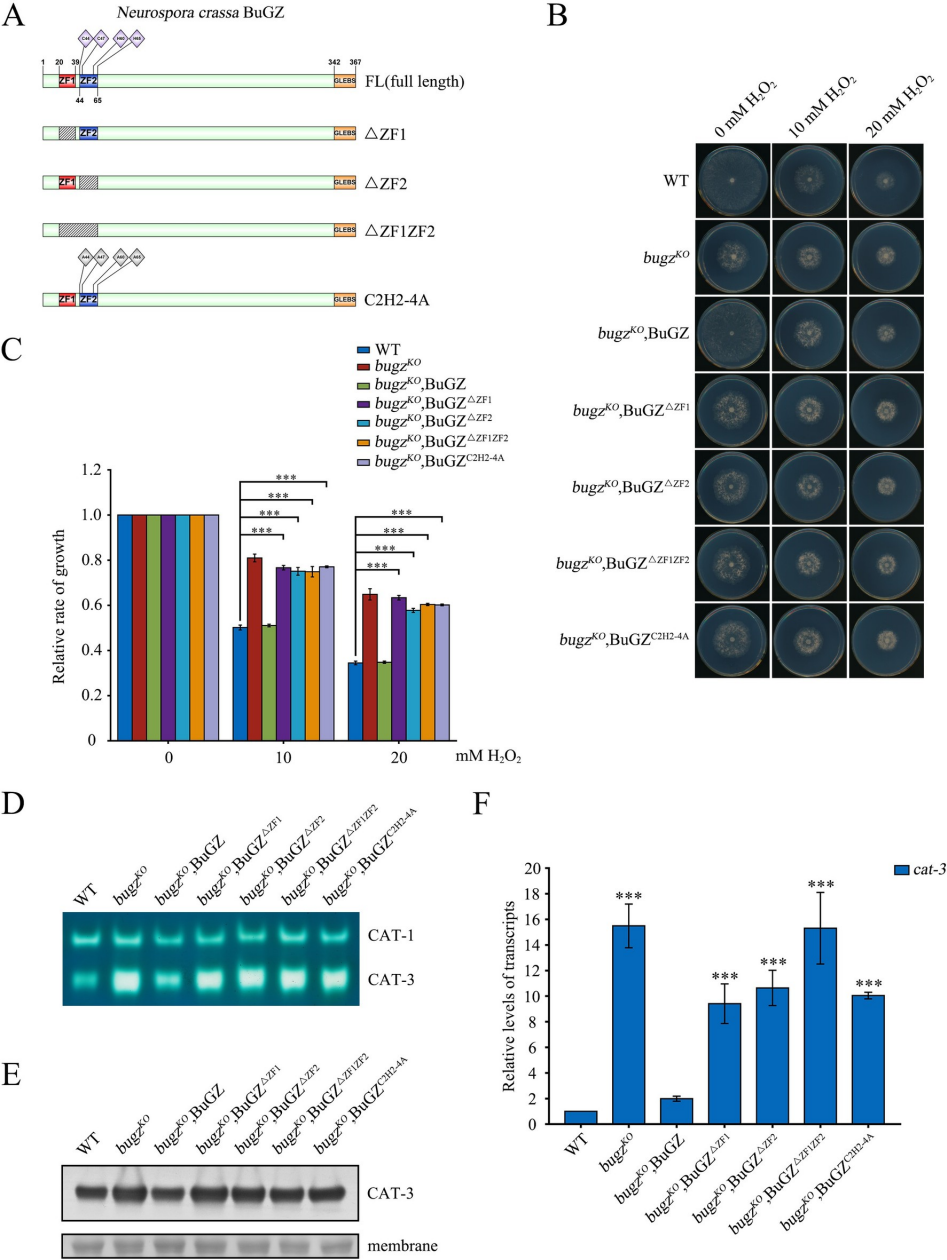
The intact zinc finger domains of BuGZ are essential for repressing *cat-3* expression. (A) Schematic of full-length BuGZ and mutants with deletions or alanine substitutions (indicated by gray diamonds) in zinc finger domains. The position of zinc finger 1 (red rectangle), zinc finger 2 (blue rectangle), GLEBS domain (yellow rectangle) and zinc ion binding sites (light purple diamonds) are indicated. (B) Mycelial growth of WT, *bugz*^*KO*^, *bugz*^*KO*^,BuGZ and transformants with various mutations in zinc finger domains on plates with 0, 10, or 20 mM H_2_O_2_. (C) Quantitation of growth rate of strains described in panel B relative to WT. (D) In-gel catalase activity assay of protein extracts from WT, *bugz*^*KO*^, *bugz*^*KO*^,BuGZ and transformants with various mutations in zinc finger domains. (E) Western blot for CAT-3 protein in WT, *bugz*^*KO*^, *bugz*^*KO*^,BuGZ and transformants with various mutations in zinc finger domains. The membranes stained by Coomassie blue served as the loading control. (F) RT-qPCR quantification of *cat-3* mRNA in *bugz*^*KO*^, *bugz*^*KO*^,BuGZ and transformants with various mutations in zinc finger domains relative to WT. Error bars indicate SD (n = 3). Significance was evaluated by two-tailed *t*-test. *P < 0.05, **P < 0.01, and ***P<0.001.

To investigate the function of the zinc finger domains of BuGZ in regulating *cat-3* expression, constructs of Myc-BuGZ with mutations at the zinc ion binding sites (C2H2-4A) or with one or both zinc finger domains deleted were expressed in the *bugz*^*KO*^ strain (Fig 6A). Plate assays showed that none of the transformants, with the exception of the Myc-tagged wild-type BuGZ restored the H_2_O_2_-resistant phenotype of *bugz*^*KO*^ strains to the level of the WT strain (Fig 6B and 6C). Furthermore, expression of Myc-BuGZ, but not zinc finger mutants, repressed expression of CAT-3 protein and *cat-3* mRNA in the *bugz*^*KO*^ strain (Fig 6D–6F). These results indicate that both zinc fingers of BuGZ are essential for the transcriptional repression of *cat-3*. However, ChIP assays performed using anti-BuGZ antibody revealed that Myc-BuGZ with mutations of the zinc finger domains showed only a slight reduced enrichment at the *cat-3* promoter (S8A Fig) and TSS region (S8B Fig) compared to the wild-type BuGZ, which probably resulted from the small reduction of BuGZ protein level in zinc finger mutants (S8C Fig) without impairing Bub3-BuGZ interaction (S8D Fig). Thus, the zinc fingers of BuGZ play other more important roles besides regulating its DNA binding to achieve *cat-3* repression.

### Increased recruitment of transcription activators in the absence of BuGZ facilitates assembly of transcription machinery on the *cat-3* chromatin

**Fig 7:**
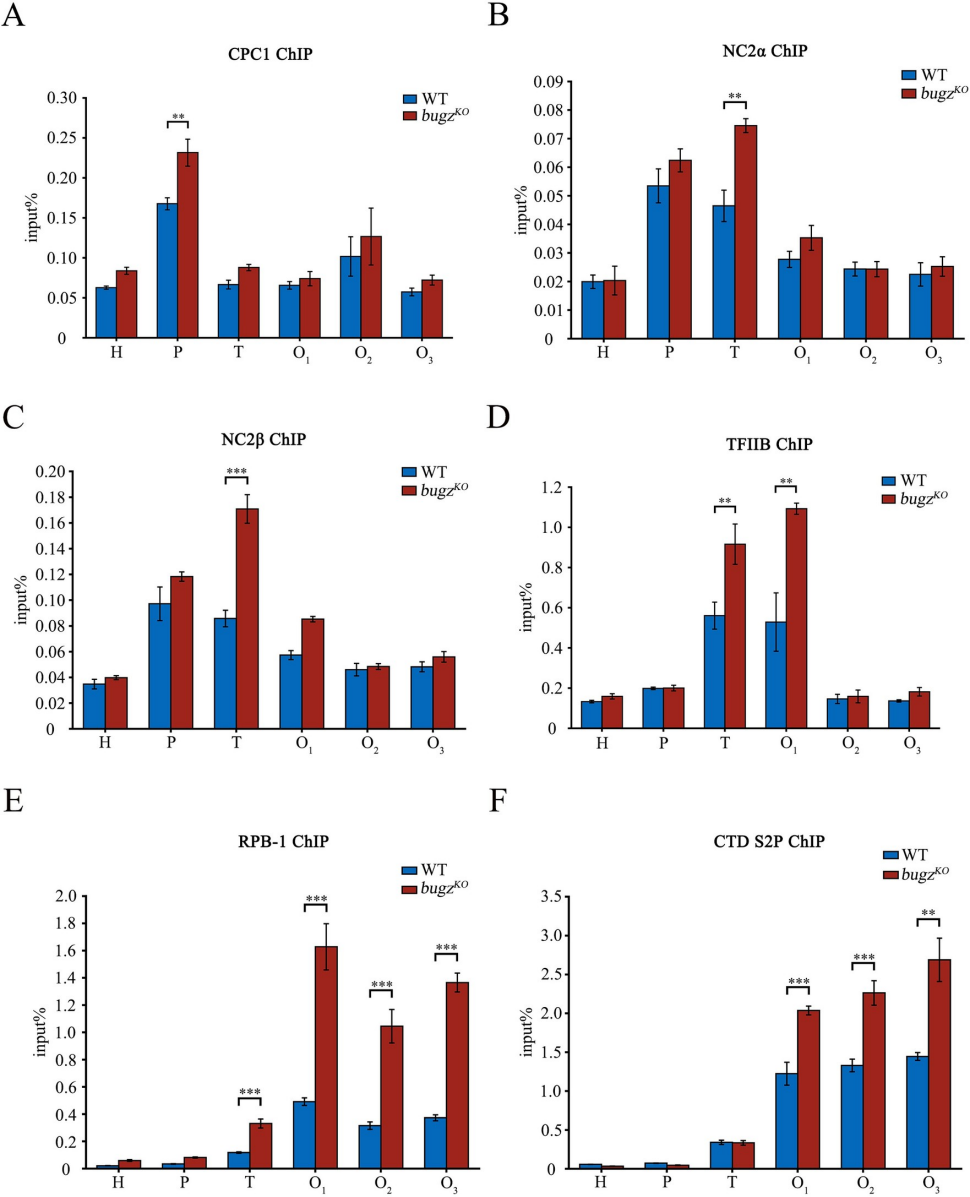
BuGZ antagonizes the recruitment of transcription activators and RNAPII to the *cat-3* gene. ChIP analyses of binding of (A) CPC1, (B) NC2α, (C) NC2β, (D) TFIIB, (E) RPB-1, and (F) RNAPII phosphorylated at Ser2 at the *cat-3* locus in WT and *bugz*^*KO*^ strains.

We previously identified CPC1/GCN4 and NC2 complex as activators of *cat-3* transcription that directly bind to the promoter or transcription start site (TSS) of the *cat-3* gene [21,22]. As our data showed that BuGZ is recruited to the *cat-3* gene region (Fig 5B), we wondered whether its transcriptional repression of *cat-3* results from blocking the recruitment of activators through steric hindrance. To test this hypothesis, we evaluated the occupancy of CPC1 and two subunits of NC2 complex (NC2α, NC2β) at the *cat-3* locus in *bugz*^*KO*^ strains by ChIP using anti-CPC1, anti-NC2α, and anti-NC2β antibodies, respectively. As expected, the CPC1 and NC2 complex were detected at the *cat-3* promoter (P) or the transcription initiation region (T) in the WT strains, but the lack of BuGZ promoted further accumulation of these factors on the *cat-3* regulatory regions (Fig 7A–7C).

Consistent with the elevated binding of CPC1 and NC2 at the *cat-3* locus in the absence of BuGZ, the recruitment of the general transcription factor TFIIB was also increased at the 5’ end of *cat-3* gene in the *bugz*^*KO*^ strain compared to the WT strain (Fig 7D), indicating more efficient assembly of the pre-initiation complex of the transcriptional machinery due to the loss of BuGZ. The level of Rpb1, which is the large subunit of RNA polymerase II (RNAPII), was also dramatically increased from the TSS throughout the open reading frame of *cat-3* gene in the *bugz*^*KO*^ strain compared to the WT strain (Fig 7E). In addition, the efficiency of RNAPII elongation, as indicated by the binding level of Ser2 phosphorylated RNAPII, was also increased inside the *cat-3* gene body in the *bugz*^*KO*^ strain compared to the WT strain (Fig 7F). These results indicate that in the absence of BuGZ, assembly of the transcriptional machinery on the *cat-3* gene and progression of its transcription are enhanced. Taken together, our data suggest that BuGZ chromatin binding at the *cat-3* gene locus interferes with binding of transcription activators to repress its expression.

## Discussion

Catalases are indispensable enzymatic antioxidants that maintain redox homeostasis in aerobic organisms. Transcription of the genes encoding these enzymes is under strict control to maintain redox homeostasis. We previously identified several transcription activators of *cat-3* expression in *N*. *crassa*, including GCN4/CPC1 and NC2 complex [21,22]. In this study, we sought to determine the mechanism responsible for the transcriptional repression of these genes. We found that deletion of the gene encoding for the mitotic proteins Bub3 or BuGZ leads to robust *cat-3* gene activation and strong resistance to H_2_O_2_ treatment in *N*. *crassa*. By constructing *bub3*^*KO*^*bugz*^*KO*^ double mutants, we discovered that Bub3 and BuGZ function in the same pathway to repress *cat-3* transcription. Interestingly, although there is a strong interaction between Bub3 and BuGZ, which is important for Bub3 stability and mitotic progression in higher eukaryotes [34, 35], our data showed that the Bub3-BuGZ interaction in *N*. *crassa* was neither required for the stability of either protein nor for the regulation of *cat-3* expression. However, the significantly reduced BuGZ levels in *bub3*^*KO*^ mutants resulted in overexpression of *cat-3*, and ectopic expression of BuGZ or BuGZ^ΔGLEBS^ both down-regulated *cat-3* transcription in the absence of Bub3, confirming BuGZ as the downstream one for *cat-3* repression. Unlike Bub3 which lacks DNA binding ability, BuGZ can directly bind to the *cat-3* gene but independent of its highly conserved zinc finger domains. More importantly, BuGZ could response to the oxidative stress signals for repressing the overexpression of *cat-3* specifically. ChIP assays revealed that loss of BuGZ makes the local chromatin at *cat-3* more accessible to transcriptional activators which results in more efficient assembly of preinitiation complex and high elongation rate of transcription. Taken together, our study uncovered the original relationship between Bub3 and BuGZ in *N*. *crassa* that is crucial to transcriptional repression of *cat-3* gene, and established the mechanism for how these mitotic proteins could directly regulate gene transcription.

Phylogenetic analysis previously revealed the differences in the evolutionary conservation of Bub3 and BuGZ [35]. Bub3 is highly conserved in all eukaryotes including budding and fission yeasts where it was first identified [28]; however, BuGZ orthologs are not found in yeast despite its strong conservation in higher eukaryotes [57]. Considering the evolutionary stage of fungi in the organic sphere, we assume that BuGZ function has undergone major changes. Evidence for a Bub3-BuGZ interaction in model organisms was revealed by examination of protein-protein interaction databases [58–63]. Our findings confirmed this interaction in *N*. *crassa*, demonstrating that it is highly conserved in eukaryotes. However, the regulatory relationship between Bub3 and BuGZ in fungi is different from that in higher eukaryotes. In human cell lines and *Xenopus* egg extracts, the interaction is necessary for Bub3 stability and kinetochore loading during mitosis [34,35]. In *N*. *crassa*, the deficiency of BuGZ did not affect Bub3 stability, whereas the lack of Bub3 caused a significant down-regulation of BuGZ protein levels. In *N*. *crassa*, the mRNA levels of *bugz* remain unchanged upon deletion of *bub3*, which is similar to the situation in human cell lines [35]. Previous studies in HeLa cells demonstrated that only two highly conserved glutamates in GLEBS domain of BuGZ are essential for the interaction between Bub3 and BuGZ [34]. Analysis of the BuGZ mutants revealed that these glutamates are necessary for this interaction in *N*. *crassa* as well. In cells with an EE to AA mutation or with an EE deletion, levels of the BuGZ was considerably reduced; however, deletion of different segments in the GLEBS domain results in loss of Bub3-BuGZ interaction but diversity of BuGZ protein stability. These data suggest that the Bub3-BuGZ interaction and BuGZ stability are regulated by different mechanisms.

The negative effect of BuGZ on gene transcription was previously reported. For instance, BuGZ was identified as a factors that negatively regulates human *NFκB1* expression [64], and a recent study performed in rice demonstrated that the BuGZ homolog acts as a transcriptional repressor of green revolution gene *SD1/OsGA20ox2* [50]. BuGZ has also been reported to activate transcription. A systematic RNAi screen in *C*. *elegans* revealed that BuGZ-1 promotes synaptic vesicle recycling by regulating the activation of endocytosis-related genes such as *rab11*.*1* [48]. Furthermore, a high-throughput analysis in human embryonic stem cells identified 177 direct target genes were up-regulated and 357 direct target genes were down-regulated in response to BuGZ depletion, with the strongest binding clustered around transcription start sites [49]. In this study, we demonstrated that the specifically binding of BuGZ is crucial for transcription repression of *cat-3* by preventing the assembly and progression of the transcription machinery. Although the highly conserved zinc fingers are almost nonfunctional for DNA binding of BuGZ, they are essential for *cat-3* repression. Given that there are no reports on the transcriptional function of BuGZ’s zinc fingers, we speculate that it may associate with some transcription-related effectors to achieve gene repression, which has been widely studied in higher organisms [65,66].

Interestingly, at the beginning we considered that high expression of *cat-3* gene corresponded to low levels of BuGZ binding. However, by introducing the oxidative stresses which induced high expression of *cat-3*, we found that the recruitment of BuGZ to *cat-3* locus further increased. Based on our data, we support a model that the *cat-3* overexpression under stimulation alters the chromatin accessibility for elevated BuGZ binding, which in turn restores chromatin to a compacted state after removal of stimuli. Collectively, we conclude that BuGZ may serve as a founder protein for recruitment of chromatin modelers and antagonize activators bindings to restrict high *cat-3* expression in normal growth condition. Whereas under oxidative stress, more open chromatin state leads to rapid response of prepared BuGZ for increased recruitment and effectively repression of *cat-3* transcription, maintaining the ROS balance to physiological homeostasis.

## Materials and methods

### Strains and culture conditions

The 87–3 (*bd*, *a*) and 4200 (*nbd*, *a*) strain were used as the *N*. *crassa* wild-type strains in this study [51]. The *ku70*^*RIP*^ (*bd*, *a*) strain, generated previously [67], was used as the recipient strain for creating the *bub3* (NCU09744) and *bugz* (NCU06145) knock-out mutants, *cat-3*^*KO*^ strain and the *bugz*^*KO*^*bub3*^*KO*^, *bub3*^*KO*^ *cat-3*^*KO*^ or *bugz*^*KO*^ *cat-3*^*KO*^ double mutants. In all cases, entire open reading frames were replaced with resistance screening genes through homologous recombination according to a protocol described previously [68]. Plasmids encoding *bub3* or *bugz* driven by the *qa-2* promoter (pqa-5Myc-6His-Bub3, pqa-5Myc-6His-BuGZ) were transformed into *bub3*^*KO*^ or *bugz*^*KO*^ (*his-3*) strains, respectively, to construct *bub3*^*KO*^,Myc-Bub3 or *bugz*^*KO*^,Myc-BuGZ complementary transformants. The plasmid pqa-5Myc-6His-BuGZ was used as the template for mutagenesis, and the mutants (BuGZ^EE-AA^, BuGZ^ΔEE^, BuGZ^ΔGLEBS^, BuGZ^ΔG1~ΔG5^, BuGZ^ΔZF1^, BuGZ^ΔZF2^, BuGZ^ΔZF1ZF2^, BuGZ^C2H2-4A^) were introduced into *bugz*^*KO*^ strains to generate corresponding mutant transformants (*bugz*^*KO*^,Myc-BuGZ^EE-AA^, *bugz*^*KO*^,Myc-BuGZ^ΔEE^, *bugz*^*KO*^,Myc-BuGZ^ΔGLEBS^, *bugz*^*KO*^,Myc-BuGZ^ΔG1~ΔG5^, *bugz*^*KO*^,Myc-BuGZ^ΔZF1^, *bugz*^*KO*^,Myc-BuGZ^ΔZF2^, *bugz*^*KO*^,Myc-BuGZ^ΔZF1ZF2^, *bugz*^*KO*^,Myc-BuGZ^C2H2-4A^). Similarly, plasmids pqa-5Myc-6His-BuGZ and pqa-5Myc-6His-BuGZ^ΔGLEBS^ were transformed into the *bub3*^*KO*^ strain to construct *bub3*^*KO*^,Myc-BuGZ and *bub3*^*KO*^,Myc-BuGZ^ΔGLEBS^ transformants, respectively.

The solid medium for plate assays contained 1× Vogel’s salts, 0.1% glucose, 0.17% arginine, 50 ng/mL biotin, and 1.5% (w/v) agar with or without H_2_O_2_ and in the absence or presence of 1x10^-3^ M QA. The slant medium contained 1× Vogel’s salts, 3% sucrose, and 1.5% (w/v) agar. Liquid cultures were grown at 25°C with shaking in minimal medium (1× Vogel’s salts and 2% glucose) for 18 h in constant light. When QA was added to liquid cultures, cultures were grown in low-glucose medium (1× Vogel’s salts, 0.1% glucose, 0.17% arginine) with 1x10^−2^ M QA.

### Plate assay

The medium for plate assays contained 1× Vogel’s salts, 0.1% glucose, 0.17% arginine, 50 ng/mL biotin, and 1.5% (w/v) agar with different concentrations of H_2_O_2_. When QA was added, 0.1% glucose was replaced with 1x10^-3^ M QA.

About 7-day-old conidia of specific strains were inoculated in petri dishes with 50 mL liquid medium containing 1× Vogel’s salts and 2% glucose under static culture condition at 25°C for 1–2 days. Disks of mycelium film were cut with a cork borer and placed in petri dishes. WT or mutant strains were inoculated at the center of the disks and grown under constant light at 25°C on the medium containing 0, 10, or 20 mM H_2_O_2_. When the WT strain almost completely covered the medium without H_2_O_2_, all the plates were scanned, and the average growth rate of each strain relative to that in medium without H_2_O_2_ (0 mM) was calculated. Each experiment was performed at least three times independently.

### Protein analyses

Protein extraction, quantification and western blot analyses were performed as described previously [69]. Equal amounts of total protein (40 μg) of different samples were loaded into distinct lanes on prepared SDS-polyacrylamide gels. After electrophoresis, proteins were transferred onto PVDF membranes. Western blot analyses were performed using antibodies against the proteins of interest.

### In-gel assay for catalase activity

Cell extracts from disks of mycelium film cultivated in liquid culture for 18 h were used for the in-gel catalase activity assays as described previously [70,71]. Equal amounts of total protein (25 μg) of different samples were loaded into distinct lanes on prepared 7.5% native-polyacrylamide gels. After electrophoresis, the gel was rinsed with doubly distilled H_2_O and then immersed in 10 mM H_2_O_2_ with gentle shaking for 10 min. The gel was then transferred into a mixture of freshly prepared 1% potassium hexacyanoferrate (III) and 1% iron (III) chloride hexahydrate. With gentle shaking for 1–2 min, catalase activity was visualized as a band where H_2_O_2_ was decomposed by catalases.

### RNA analyses

For the RT-qPCR, total RNA was isolated with TRIzol agent containing 38% phenol in 0.8 M guanidine thiocyanate, 0.4 M ammonium thiocyanate, 0.1 M sodium acetate (pH 5.0), and 5% glycerol. Each RNA sample (total RNA, 5 μg) was treated with a double-strand specific DNase to remove contaminating genomic DNA and then subjected to reverse transcription with the Maxima H Minus cDNA Synthesis Master Mix containing Maxima H Minus Reverse Transcriptase and Thermo Scientific RiboLock RNase Inhibitor (Thermo Scientific M1682). Finally, the cDNA products were amplified by real-time PCR. The primers used for qPCR are listed in S1 Table. The relative values of gene expression were calculated using the 2^−ΔΔCT^ method by comparing the cycle number of each sample with that of the untreated control [72]. The results were normalized to the expression levels of *β-tubulin* gene.

### Generation of antibodies against Bub3 and BuGZ

GST-Bub3 (containing Bub3 amino acids M115-L263) and GST-BuGZ (containing BuGZ amino acids A191-P352) fusion proteins were expressed in *Escherichia coli* BL21 cells, and the soluble recombinant proteins were purified and used as the antigens to immunize rabbits to generate rabbit polyclonal antiserum as described previously [69].

### Co-immunoprecipitation assay

Cell extracts from the mycelium cultured in liquid medium were used for co-immunoprecipitation analyses. Protein extraction and quantification were performed as described previously [69]. Extracts (total protein, 2 mg/mL) in extraction buffer (50 mM HEPES (pH7.4), 137 mM NaCl and 10% glycerol) were incubated with 2 μL of anti-c-Myc mouse monoclonal antibody (TransGen Biotech, #HT101) at 4°C with rotation. After about 4 h, 40 μL pre-equilibrated protein G beads (GE Healthcare, #17-0885-02) were added and incubated for 2 h at 4°C with rotation. Beads were then washed 6–8 times with ice-cold extraction buffer, and mixed with protein loading buffer followed by boiling for 10 min to elute the bound protein. Finally, the immunoprecipitated proteins were analyzed by western blotting.

### ChIP analyses

ChIP assays were performed as described previously [73]. In brief, tissues were fixed with 1% formaldehyde for 15 min at 25°C with shaking. Reactions were stopped by addition of glycine at a final concentration of 125 mM for 5 min. Cross-linked tissues were ground and resuspended in lysis buffer containing proteinase inhibitors, and chromatin was sheared by sonication to 500–1000 bp fragments. Samples of 2 mg/mL protein were used per immunoprecipitation reaction, and 10 μL was kept as the input DNA. The ChIP assay was carried out with 10 μL of antibody to BuGZ (self-prepared), 3 μL of antibody to H3 (CST, #2650), 2 μL of antibody to H2B (Abcam, #ab1790), 5 μL of antibody to CPC-1 (self-prepared), 8 μL of antibody to TFIIB (self-prepared), 5 μL of antibody to RPB-1 (self-prepared), and 7 μL of antibody to RNAPII phosphorylated at Ser2 (Abcam, #ab5095). Immunoprecipitated DNA was quantified using real-time PCR (ABI, 7500) with primer pairs listed in S2 Table. ChIP-qPCR data are presented as a percentage of input DNA.

## Supporting information

S1 Fig *bub3*^*KO*^ mutant is resistant to H_2_O_2_-induced oxidative stress and shows elevated *cat-3* expression. (A) Mycelial growth of WT and *bub3*^*KO*^ (*bd*) strains on plates with addition of 0, 10, or 20 mM H_2_O_2_. (B) Quantitation of growth relative to WT of *bub3*^*KO*^ (*bd*) strain under conditions described in panel A. (C) In-gel catalase activity assay of protein extracts from WT and *bub3*^*KO*^ strains. (D) The level of CAT-3 protein in WT and *bub3*^*KO*^ strains determined by western blot analyses. The membranes stained by Coomassie blue served as the loading control. (E) Levels of *cat-3* mRNA in *bub3*^*KO*^ strain relative to that in the WT strain as determined by RT-qPCR analyses. Error bars indicate SD (n = 3). Significance was evaluated by two-tailed *t*-test. *P < 0.05, **P < 0.01, and ***P<0.001.
(TIF)

S2 Fig Bub3 and BuGZ are highly conserved among eukaryotes. Amino acid sequence alignments of (A) Bub3 and (B) BuGZ from *Neurospora crassa*, *Homo sapiens*, *Mus musculus*, *Xenopus laevis*, *Danio rerio*, *Drosophila melanogaster*, *Caenorhabditis elegans*, *Schizosaccharomyces pombe*, *and Saccharomyces cerevisiae*. Seven WD40 repeats of Bub3, two zinc finger domains and the GLEBS domain of BuGZ are marked in black boxes. The zinc ion binding sites of BuGZ are indicated with red asterisks.
(TIF)

S3 Fig The H_2_O_2_-resistant phenotype of *bub3*^*KO*^ and *bugz*^*KO*^ strain was predominantly due to elevated CAT-3 activity. (A) Mycelial growth of WT, *cat-3*^*KO*^, *bub3*^*KO*^, *bub3*^*KO*^*cat-3*^*KO*^, *bugz*^*KO*^ and *bugz*^*KO*^*cat-3*^*KO*^ strains on plates with 0, 5, 10, or 20 mM H_2_O_2_. (B) Quantitation of growth relative to WT of *cat-3*^*KO*^, *bub3*^*KO*^, *bub3*^*KO*^*cat-3*^*KO*^, *bugz*^*KO*^ and *bugz*^*KO*^*cat-3*^*KO*^ strains under conditions described in panel A.
(TIF)

S4 Fig BuGZ is a negative transcriptional regulator of *cat-3* gene expression. (A) Mycelial growth of WT, *bugz*^*KO*^, and *bugz*^*KO*^,BuGZ strains on plates with 0, 10, or 20 mM H_2_O_2_. (B) Quantitation of growth relative to WT of *bugz*^*KO*^ and *bugz*^*KO*^,BuGZ strains under conditions described in panel A. (C) In-gel catalase activity in protein extracts from WT, *bugz*^*KO*^, and *bugz*^*KO*^,BuGZ strains. (D) Western blot analysis of CAT-3 protein in WT, *bugz*^*KO*^, and *bugz*^*KO*^,BuGZ strains. The membranes stained by Coomassie blue served as the loading control. (E) RT-qPCR quantification of *cat-3* mRNA relative to WT in *bugz*^*KO*^ and *bugz*^*KO*^,BuGZ strains. Error bars indicate SD (n = 3). Significance was evaluated by two-tailed *t*-test. *P < 0.05, **P < 0.01, and ***P<0.001.
(TIF)

S5 Fig Rabbit-derived polyclonal antibodies specifically recognize *N*. *crassa* Bub3 and BuGZ. Immunodetection of Bub3 or BuGZ in WT and (A) *bub3*^*KO*^ or (B) *bugz*^*KO*^ strains using polyclonal antibodies that specifically recognize Bub3 or BuGZ protein, respectively. The membranes stained by Coomassie blue served as the loading control.
(TIF)

S6 Fig Ectopic expression of BuGZ or BuGZ^ΔGLEBS^ protein reduce CAT-3 expression in *bub3* mutants. (A) In-gel catalase activity assay of protein extracts from WT, *bub3*^*KO*^, *bub3*^*KO*^,Bub3, *bub3*^*KO*^,BuGZ and *bub3*^*KO*^,BuGZ^ΔGLEBS^ strains. (B) Western blot for CAT-3 protein in WT, *bub3*^*KO*^, *bub3*^*KO*^,Bub3, *bub3*^*KO*^,BuGZ and *bub3*^*KO*^,BuGZ^ΔGLEBS^ strains. The membranes stained by Coomassie blue served as the loading control.
(TIF)

S7 Fig BuGZ negatively regulates the peroxiredoxin encoding gene *prx1* but its recruitment does not respond to MD-induced *prx1* overexpression. (A) RT-qPCR quantification of peroxiredoxin encoding gene *hyr1* and *prx1* mRNA relative to WT in *bugz*^*KO*^ and *bub3*^*KO*^ strains. (B) Levels of *cat-3* mRNA change in WT strain after H_2_O_2_ or MD treatment for 2 hours determined by RT-qPCR analyses. (C) Levels of *prx1* mRNA change in WT strain after MD treatment for 2 hours determined by RT-qPCR analyses. (D) ChIP analysis of the binding of BuGZ after MD treatment for 2 hours at *prx1* locus. Primer *prx1* P, *prx1* T, *prx1* O_1_, *prx1* O_2_ indicate the promoter, TSS and ORF regions of *prx1* respectively as marked in *cat-3* locus.
(TIF)

S8 Fig Mutations in zinc finger domains do not dramatically affect the DNA binding ability of BuGZ, as well as the stability of BuGZ and Bub3-BuGZ interaction. (A, B) ChIP analysis of the binding of Myc-BuGZ with various mutations in zinc finger domains at *cat-3* (A) promoter or (B) TSS region (the primer P and primer T are identical to those in Fig 5A). (C) Western blot analyses of the level of BuGZ protein in WT, *bugz*^*KO*^, *bugz*^*KO*^,BuGZ and transformants with various mutations in zinc finger domains. The membranes stained by Coomassie blue served as the loading control. A non-specific protein band is marked by an asterisk. (D) Co-immunoprecipitation analyses of interactions between Myc-BuGZ with various mutations in zinc finger domains and endogenous Bub3.
(TIF)

S1 Table Primers for RT-qPCR assays. (XLSX)

S2 Table Primers for ChIP-qPCR assays. (XLSX)
