# Supplementary figures and images for: A role for the mitotic proteins Bub3 and BuGZ in transcriptional regulation of catalase-3 expression
Source: PLoS Genet. 2022 Jun 6;18(6):e1010254. doi: 10.1371/journal.pgen.1010254 (PMC9203020; doi:10.1371/journal.pgen.1010254)

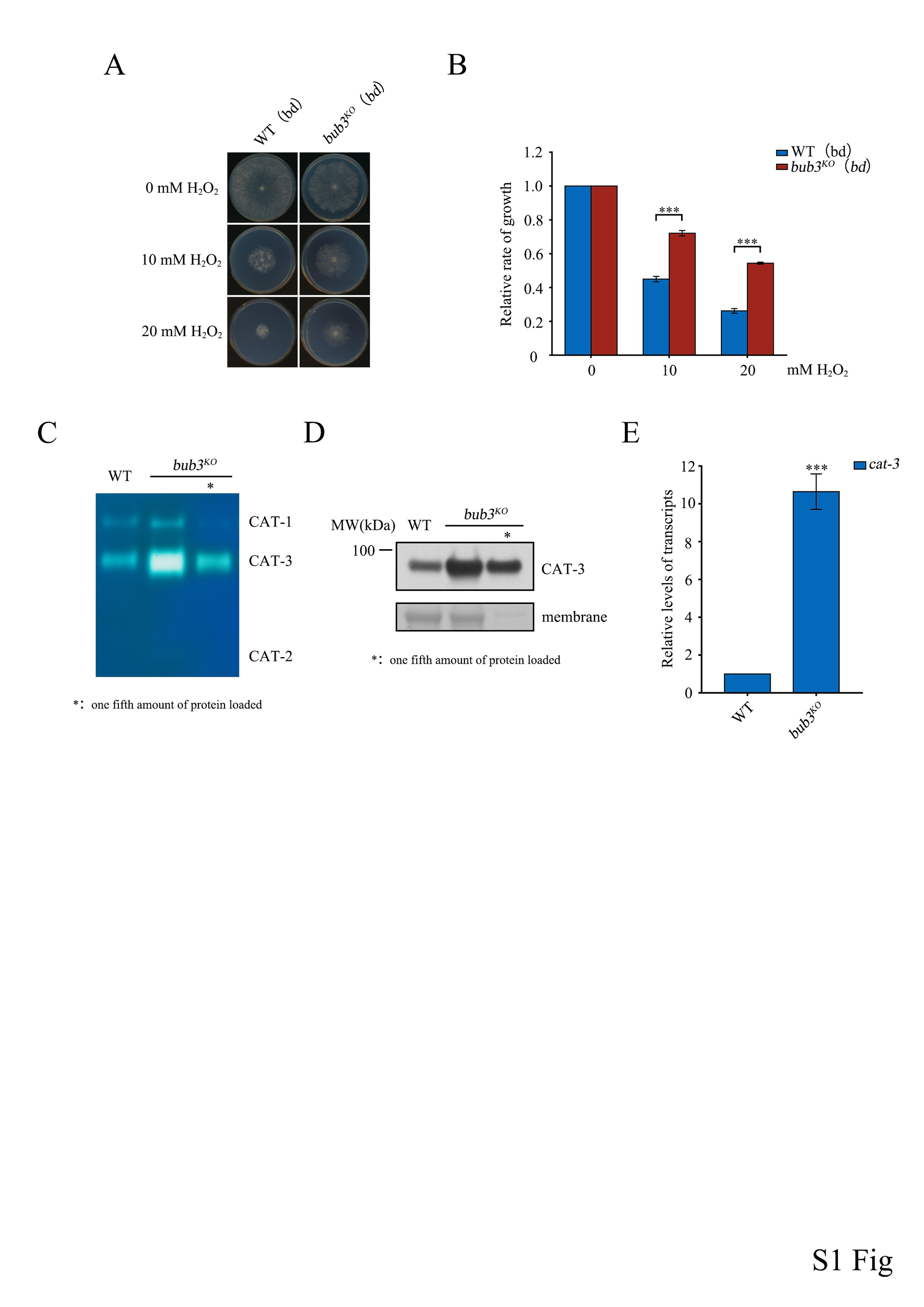

Supplement: S1 Fig — (A) Mycelial growth of WT and bub3KO (bd) strains on plates with addition of 0, 10, or 20 mM H2O2. (B) Quantitation of growth relative to WT of bub3KO (bd) strain under conditions described in panel A. (C) In-gel catalase activity assay of protein extracts from WT and bub3KO strains. (D) The level of CAT-3 protein in WT and bub3KO strains determined by western blot analyses. The membranes stained by Coomassie blue served as the loading control. (E) Levels of cat-3 mRNA in bub3KO strain relative to that in the WT strain as determined by RT-qPCR analyses. Error bars indicate SD (n = 3). Significance was evaluated by two-tailed t-test. *P < 0.05, **P < 0.01, and ***P<0.001. (TIF) [file pgen.1010254.s001.tif]

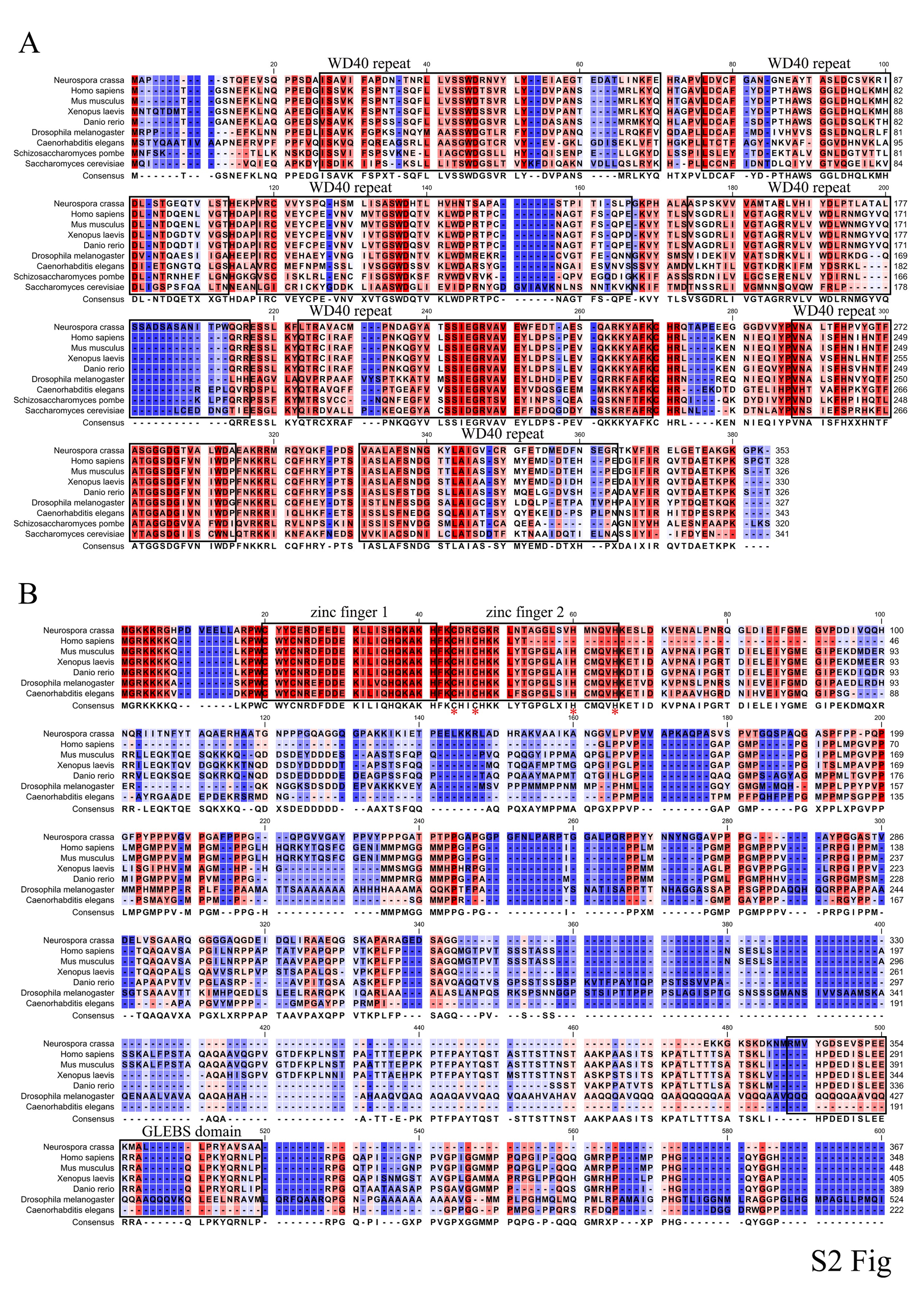

Supplement: S2 Fig — Amino acid sequence alignments of (A) Bub3 and (B) BuGZ from Neurospora crassa, Homo sapiens, Mus musculus, Xenopus laevis, Danio rerio, Drosophila melanogaster, Caenorhabditis elegans, Schizosaccharomyces pombe, and Saccharomyces cerevisiae. Seven WD40 repeats of Bub3, two zinc finger domains and the GLEBS domain of BuGZ are marked in black boxes. The zinc ion binding sites of BuGZ are indicated with red asterisks. (TIF) [file pgen.1010254.s002.tif]

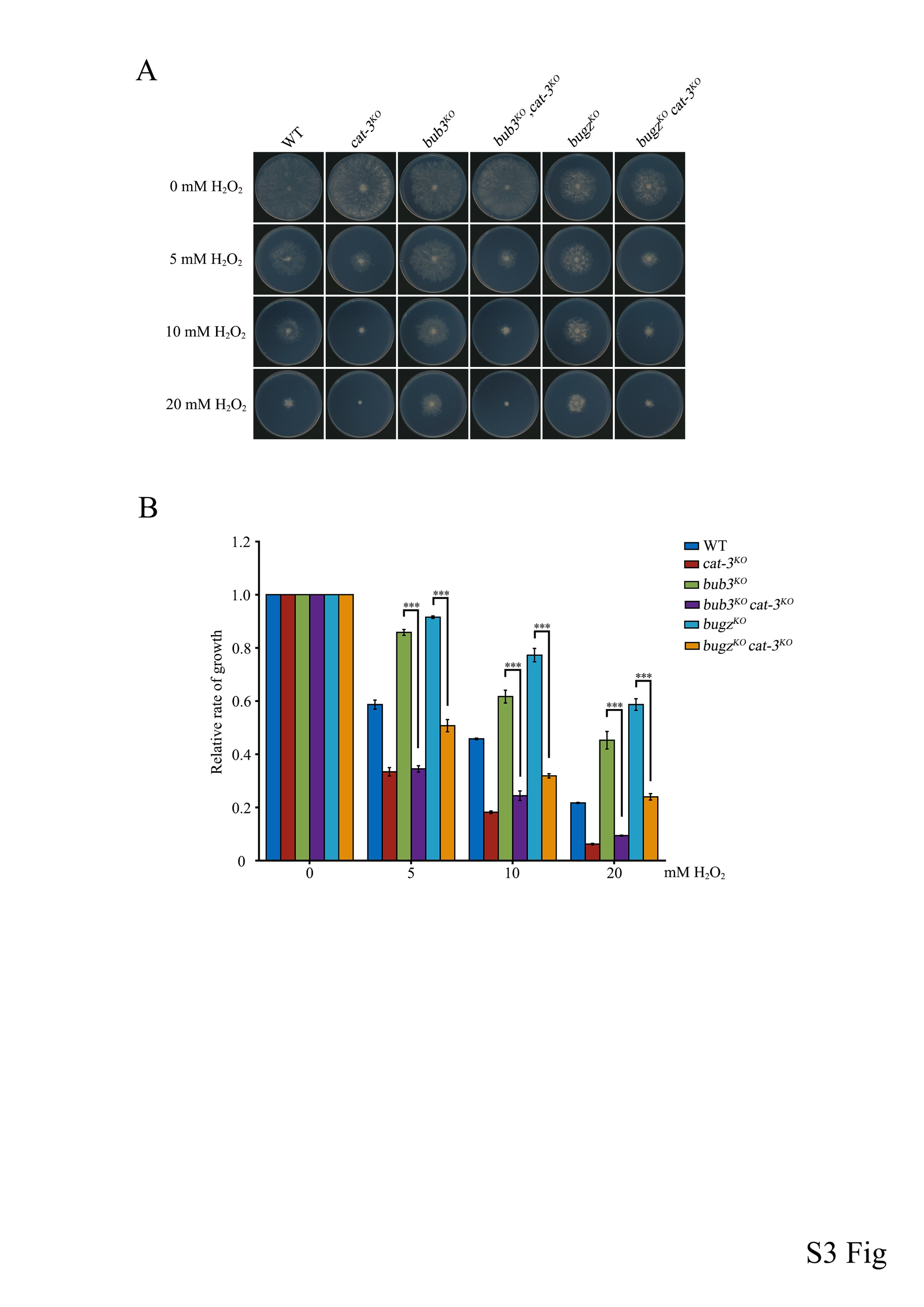

Supplement: S3 Fig — (A) Mycelial growth of WT, cat-3KO, bub3KO, bub3KOcat-3KO, bugzKO and bugzKOcat-3KO strains on plates with 0, 5, 10, or 20 mM H2O2. (B) Quantitation of growth relative to WT of cat-3KO, bub3KO, bub3KOcat-3KO, bugzKO and bugzKOcat-3KO strains under conditions described in panel A. (TIF) [file pgen.1010254.s003.tif]

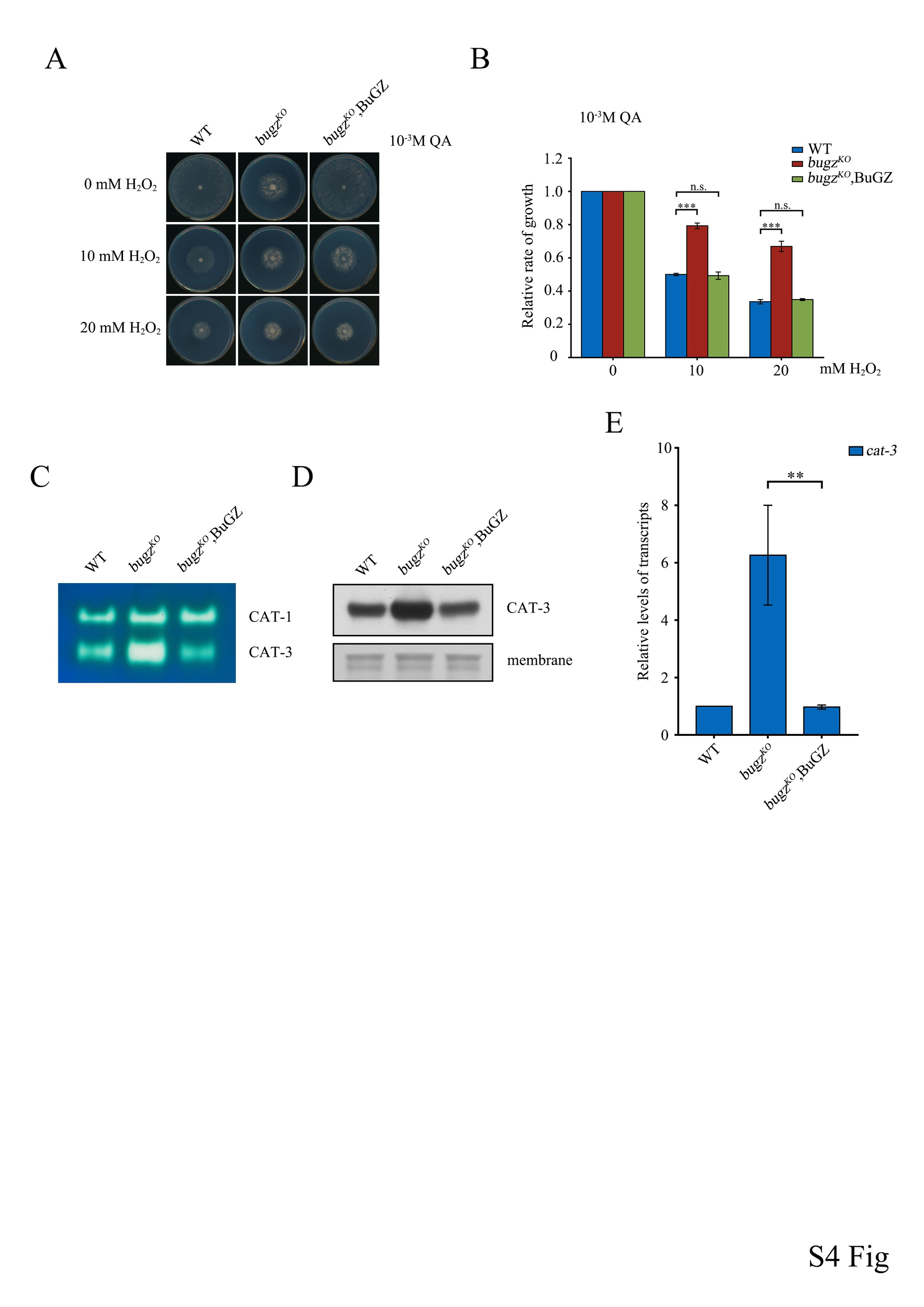

Supplement: S4 Fig — (A) Mycelial growth of WT, bugzKO, and bugzKO,BuGZ strains on plates with 0, 10, or 20 mM H2O2. (B) Quantitation of growth relative to WT of bugzKO and bugzKO,BuGZ strains under conditions described in panel A. (C) In-gel catalase activity in protein extracts from WT, bugzKO, and bugzKO,BuGZ strains. (D) Western blot analysis of CAT-3 protein in WT, bugzKO, and bugzKO,BuGZ strains. The membranes stained by Coomassie blue served as the loading control. (E) RT-qPCR quantification of cat-3 mRNA relative to WT in bugzKO and bugzKO,BuGZ strains. Error bars indicate SD (n = 3). Significance was evaluated by two-tailed t-test. *P < 0.05, **P < 0.01, and ***P<0.001. (TIF) [file pgen.1010254.s004.tif]

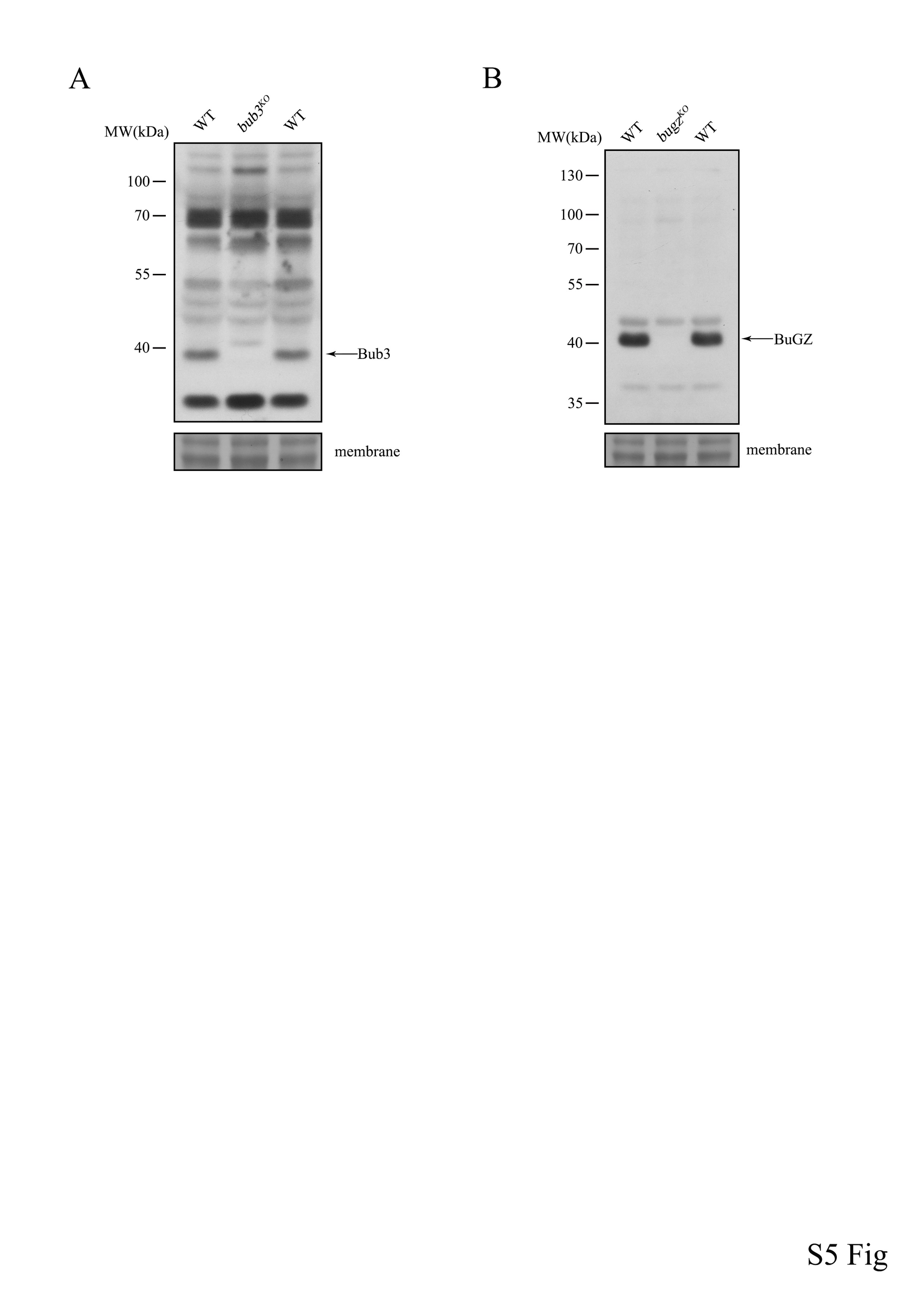

Supplement: S5 Fig — Immunodetection of Bub3 or BuGZ in WT and (A) bub3KO or (B) bugzKO strains using polyclonal antibodies that specifically recognize Bub3 or BuGZ protein, respectively. The membranes stained by Coomassie blue served as the loading control. (TIF) [file pgen.1010254.s005.tif]

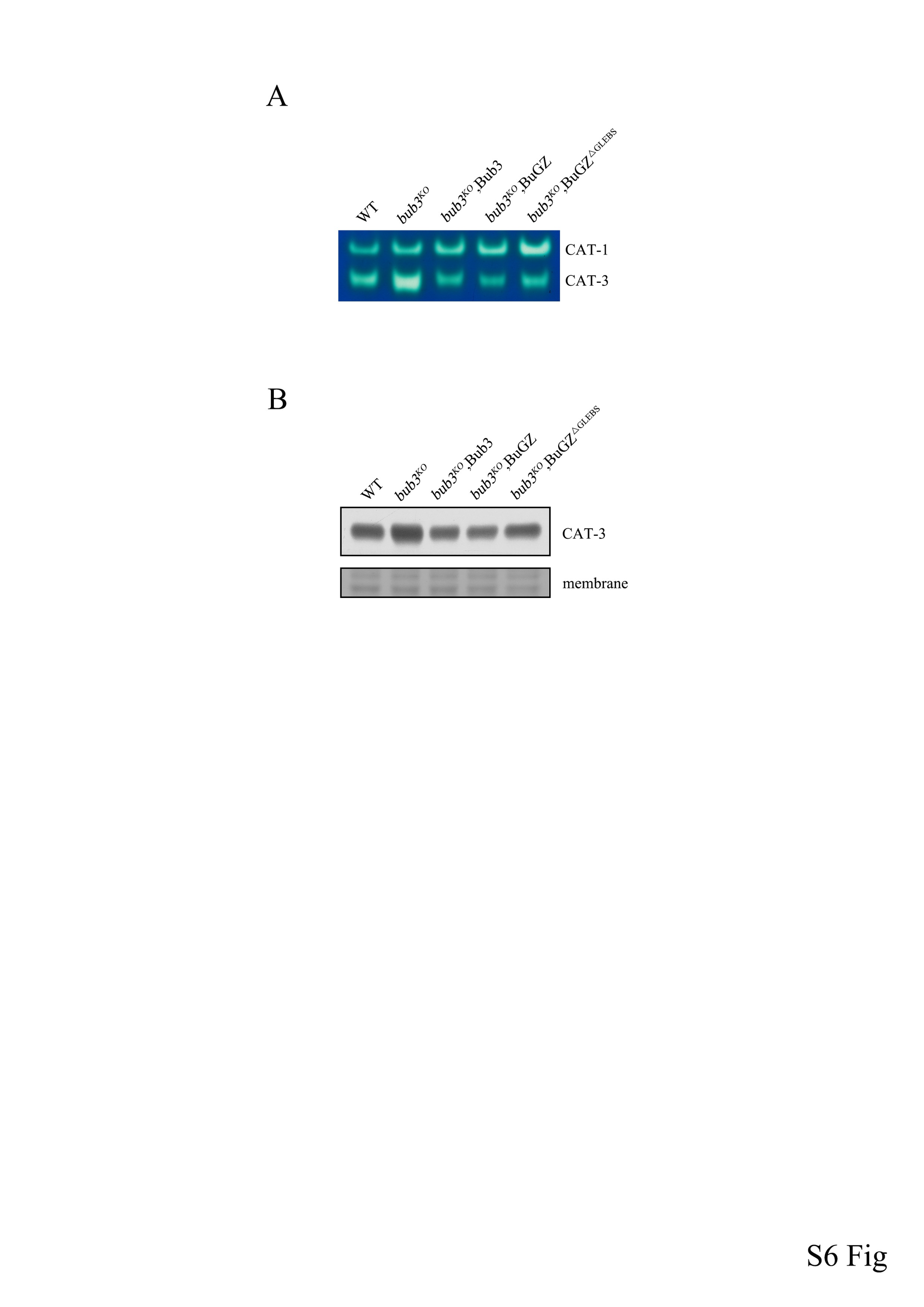

Supplement: S6 Fig — (A) In-gel catalase activity assay of protein extracts from WT, bub3KO, bub3KO,Bub3, bub3KO,BuGZ and bub3KO,BuGZΔGLEBS strains. (B) Western blot for CAT-3 protein in WT, bub3KO, bub3KO,Bub3, bub3KO,BuGZ and bub3KO,BuGZΔGLEBS strains. The membranes stained by Coomassie blue served as the loading control. (TIF) [file pgen.1010254.s006.tif]

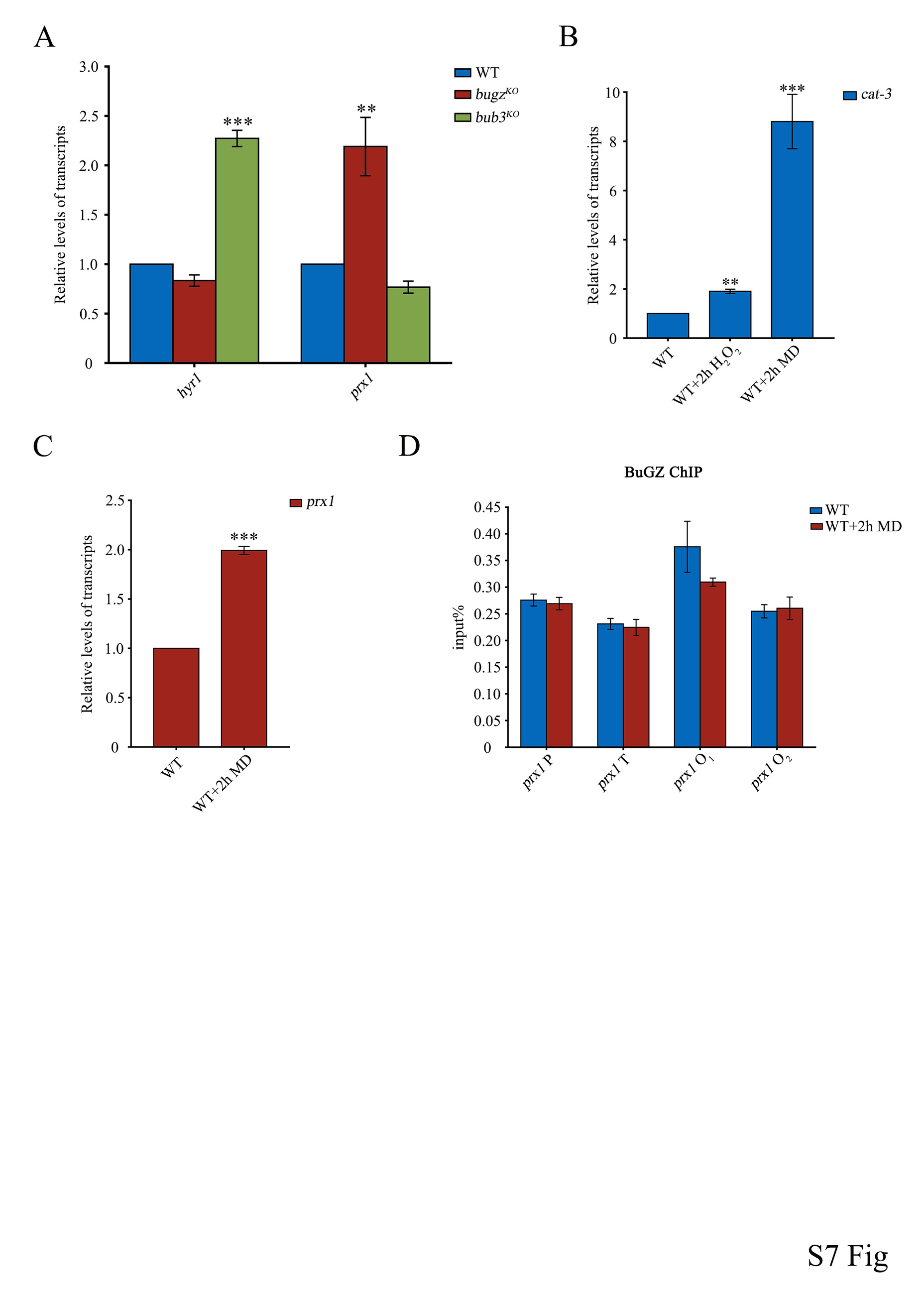

Supplement: S7 Fig — (A) RT-qPCR quantification of peroxiredoxin encoding gene hyr1 and prx1 mRNA relative to WT in bugzKO and bub3KO strains. (B) Levels of cat-3 mRNA change in WT strain after H2O2 or MD treatment for 2 hours determined by RT-qPCR analyses. (C) Levels of prx1 mRNA change in WT strain after MD treatment for 2 hours determined by RT-qPCR analyses. (D) ChIP analysis of the binding of BuGZ after MD treatment for 2 hours at prx1 locus. Primer prx1 P, prx1 T, prx1 O1, prx1 O2 indicate the promoter, TSS and ORF regions of prx1 respectively as marked in cat-3 locus. (TIF) [file pgen.1010254.s007.tif]

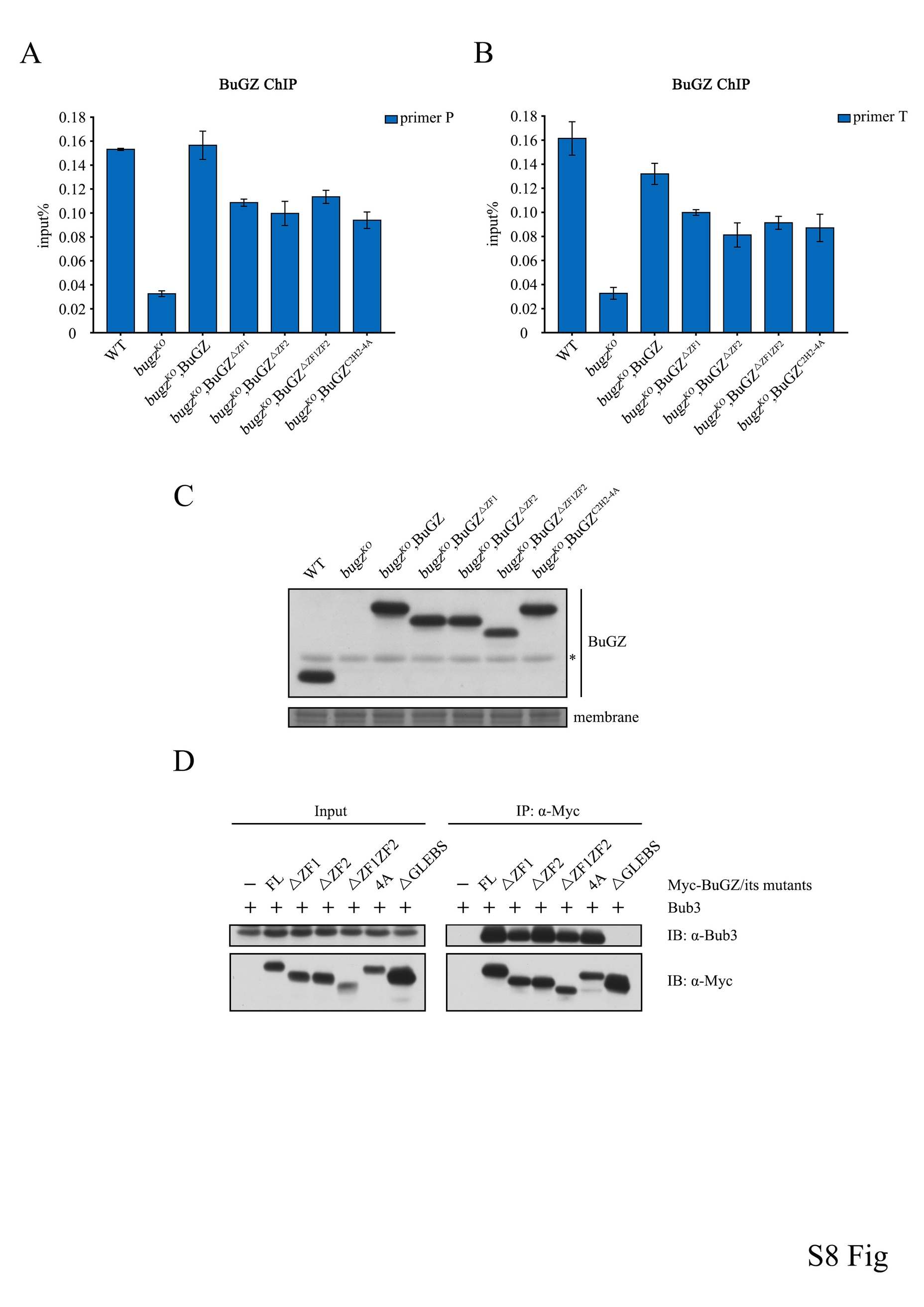

Supplement: S8 Fig — (A, B) ChIP analysis of the binding of Myc-BuGZ with various mutations in zinc finger domains at cat-3 (A) promoter or (B) TSS region (the primer P and primer T are identical to those in Fig 5A). (C) Western blot analyses of the level of BuGZ protein in WT, bugzKO, bugzKO,BuGZ and transformants with various mutations in zinc finger domains. The membranes stained by Coomassie blue served as the loading control. A non-specific protein band is marked by an asterisk. (D) Co-immunoprecipitation analyses of interactions between Myc-BuGZ with various mutations in zinc finger domains and endogenous Bub3. (TIF) [file pgen.1010254.s008.tif]
