# Supplementary material for: A role for the mitotic proteins Bub3 and BuGZ in transcriptional regulation of catalase-3 expression
Source: PLoS Genet. 2022 Jun 6;18(6):e1010254. doi: 10.1371/journal.pgen.1010254 (PMC9203020; doi:10.1371/journal.pgen.1010254)
Supplement: Attachment — Submitted filename: Cat-3 PLoS Genetics Review 2022.docx [file pgen.1010254.s011.docx]

This MS reports a novel role for proteins Bub3 and BuGZ in the transcriptional repression of the catalase gene *cat-3* in the fungus *Neurospora crassa*. In other eukaryotes Bub3 and its chaperone BuGZ have been involved in the spindle assembly checkpoint (SAC), a cell-cycle checkpoint that prevents the separation of the duplicated chromosomes until each chromosome is attached to the spindle. Therefore, the involvement of these proteins in the transcriptional regulation of a protein involved in ROS homeostasis is a novel and interesting finding. However, the authors do not clearly address weather this novel function is additional to their mitotic functions or simply these proteins play a different function (transcriptional regulation) in filamentous fungi. This should be made clear considering that the Author Summary states, “Our results not only expand the mechanism of catalase transcriptional regulation for maintaining redox homeostasis, but also imply that the cell cycle of fungi may be inextricably linked to its antioxidant processes”. This important claim can only be sustained if Bub3 and BuGZ also play a cell cycle role in *N. crassa*. Overall, the MS and the results are very clear. However, the authors need to address the following points:

Results

1. Line 124, Figure 1 and Figure 1 legend. WT strains are not labelled as bd or not bd in Figure 1. Colony diameter should be used in Figures 1B, D and similar figures, as actual growth rate was not determined in these experiments. Can the authors discuss why “the rescue phenotype was only seen when the bub3KO was in the nonband (nbd) background which has a wild-type ras-1 gene [50]. Thus, subsequent experiments related to bub3KO transformants were carried out in this genetic background”?

2. Lines 146-148. Increased CAT-3 activity in bub3KO (*nbd*) mutant correlates with increased H_2_O_2_-resistance but does not confirm (prove) that H_2_O_2_-resistant phenotype observed in the mutant was “predominantly due to elevated CAT-3 activity”. There are other enzymes important for H_2_O_2_ detoxification such as the peroxiredoxins. It is therefore essential to perform the experiments in Figure 1, using a bub3KO (*nbd*) mutant lacking the *cat-3* gene. The results could drastically change some of the conclusions of this MS.

3. Lines 186-191. The lack of Bub3 led to a considerable reduction of BuGZ protein levels and a bugzKO bub3KO double mutant show the same effects on CAT-3 expression. This suggests that bub3KO phenotype is due to BuGZ decreased levels. Yet, the phenotypes of the null mutants are so different in terms of growth and morphology. The authors need to discuss this. Is it possible that Bub3 and BuGZ do not work on the spindle assembly checkpoint (SAC) pathway in *N. crassa*?

4. Lines 255-257 and Fig. 7G. The reduced enrichment of Myc-BuGZ with mutations of the zinc finger domains at the *cat-3* promoter are very minor compared to the wild-type BuGZ, while the effects on CAT-3 activity are very clear. Any comment on this?

Discussion

5. The authors state: “In agreement with reports that elevated ROS levels promote conidiation observed in the cat-3KO and ras-1bd strains [20, 50], we observed deficiencies in mycelial growth rates, hyphal formation, and conidial development in the bub3KO and bugzKO strains corresponding to the low levels of ROS caused by elevated CAT-3 expression”. However, ROS levels were not actually measured. More importantly, to prove this important point is necessary to perform the experiments using bub3KO and bugzKO mutants lacking the *cat-3* gene. In addition, the differences in mycelial growth rates, hyphal formation, and conidial development between bub3KO and bugzKO mutants were not addressed in this MS (for example no comments were made on Fig. 3A), and conidiation levels were not determined.

Minor

Peroxiredoxins are not mentioned in lines 65-67.

References on ROS signaling (1 and 4-6) are too old (outdated).

Reference 40 cited as evidence of “Bub3 and BubGZ being implicated in the occurrence of cancer” seems contradictory with the title and content of the reference: “Germline mutations in the spindle assembly checkpoint genes BUB1 and BUB3 are infrequent in familial colorectal cancer and polyposis”.

Line 264. “As our data”.
