# Supplementary material for: A role for the mitotic proteins Bub3 and BuGZ in transcriptional regulation of catalase-3 expression
Source: PLoS Genet. 2022 Jun 6;18(6):e1010254. doi: 10.1371/journal.pgen.1010254 (PMC9203020; doi:10.1371/journal.pgen.1010254)
Supplement: Attachment — Submitted filename: Response to reviewers.docx [file pgen.1010254.s012.docx]

Dear Editors and Reviewers:

Thank you very much for your letter and for the reviewers’ comments concerning our manuscript entitled **"A role for the mitotic proteins Bub3 and BuGZ in transcriptional regulation of *catalase-3* expression" (PGENETICS-D-21-01690).** Those comments are all valuable and very helpful for revising and improving our paper, as well as the important guiding significance to our researches. We have studied comments carefully and have made correction which we hope meet with approval. Revised portion are marked in red in the paper. The main corrections in the paper and the responds to the reviewers’ comments are as following. We hope the manuscript is acceptable for publication now.

Thank you very much for your consideration.

Sincerely,

Qun He

Professor

State Key Laboratory of Agrobiotechnology,

College of Biological Sciences,

China Agricultural University,

Beijing, 100193, China

Email: qunhe@cau.edu.cn

Tel: +86-10-62731206

Fax: +86-10-62731332

The reviewers appreciated the novelty of the findings, as did I. However, the two key questions that need to be addressed are:

1. How does BUGZ bind to or associate with DNA? You will see that Rev2 (point 4) and Rev3 (point 3) both mention his issue, and I reviewed Figure 7 carefully as well. The impact of the ZNF mutations on ChIP outcome seems marginal, especially when compared with a BUGZ deletion mutant in Figure 6. I share the reviewers' concern that the DNA interaction is nonspecific. You need to examine a control gene regulatory region for ChIP binding. These would be genes whose expression is unaffected by a BUGZ deletion. Note that simply toning down the conclusion as recommended by Rev 3 would not make the manuscript acceptable at PLOS Genetics, because the level of the advance would not be sufficient.

Response: Special thanks to the editors and reviewers for affirmation and appreciation of the novelty of our manuscript. According to reviewer’s suggestion, we selected two genes encoding peroxiredoxins (*hyr-1* and *prx1*) in *N. crassa* to check their expression changes by RT-qPCR analyses in WT, *bugz^KO^*, and *bub3^KO^* strains (S7A Fig) and examined the binding of BuGZ at their promoter regions by ChIP assay as positive or negative control for BuGZ binding (Fig 5B), which confirmed the specificity of BuGZ on DNA binding and regulatory function. We also examined the BuGZ binding on a telomeric heterochromatin region for another negative control (Fig 5B). In addition, we found the increased binding of BuGZ to *cat-3* locus responds to oxidative stresses by H_2_O_2_ or menadione treatment (Fig 5C and 5D), indicating the functional specificity of BuGZ on *cat-3* gene. The manuscript of this part has been revised in the fourth section of the Result.

Considering the reviewer’s suggestion, we inspected our BuGZ ChIP data on the impact of zinc fingers mutation carefully and decided to change the conclusion that the zinc fingers of BuGZ play other more important roles besides regulating its DNA binding to achieve *cat-3* repression.

2. Rev2 (point 2) mentions a concern that I share, which is that *cat-3* may not be the sole relevant target of BuGZ. Please follow the reviewers' suggestion or test expression of other relevant genes, such as those encoding peroxiredoxins.

Please note as well that the promoter region used for ChIP assays in Figure 7 is not indicated in the legend, unlike the situation with Figure 6. Please attend to that.

Response: According to the reviewers’ suggestion, we created the *bub3^KO^cat-3^KO^* and *bugz^KO^cat-3^KO^* double mutants and examined their H_2_O_2_-resistant phenotype. Plate assay showed that lack of *cat-3* gene results in greatly enhanced sensitivity of *bub3^KO^* or *bugz^KO^* strains to H_2_O_2_, even comparable to that of *cat-3^KO^* strains (S3 Fig). Therefore, we confirmed that the H_2_O_2_-resistant phenotype observed in either single mutant was “predominantly due to elevated CAT-3 activity”.

To follow the suggestion from editors and reviewers, we detected the expression of two peroxiredoxin genes (*hyr1* and *prx1*) in *N. crassa* in WT, *bugz^KO^* or *bub3^KO^* strains. RT-qPCR analyses showed that BuGZ could negatively regulated *prx1* gene but has no effect on *hyr1* expression. In contrast, Bub3 negatively regulated *hyr1* but not *prx1* expression (S7A Fig). Thus, it is true that *cat-3* is not the sole relevant target of BuGZ. Nonetheless, other related genes have little effect on H_2_O_2_-resistance (S3 Fig).

We have labelled the promoter region primer used for ChIP assays in Fig 7 legend.

Responds to the reviewers’ comments:

Reviewer #1:

In this manuscript, the authors unraveled a novel function for proteins better known as involved in spindle assembly, namely transcription regulation, as well as providing details of the likely underlying mechanism. They investigated the role of the spindle assembly checkpoint proteins Bub3 and BuGZ in the transcriptional regulation of the *cat-3* gene in the filamentous fungus *Neurospora crassa*. They found that the proteins negatively regulate *cat-3* transcription, depending particularly on BuGZ levels and its DNA binding and not on BuGZ/Bub3 interactions. This DNA binding hampers the recruitment of transcription activators required for transcription. The authors also compare the situation in different organisms. The manuscript is well and concisely written and nice to read.

Minor point: I wonder if Figs. 1A and 1B are really required, because the same information is shown in Figs. 1C and 1D, respectively. The same applies to Figs. 2A, 2B and 2C, with the information repeated in Figs. 2C, 2D and 2F, respectively.

Response: Thanks for reviewer’s comments. To follow the reviewer’s suggestion, we have retained initial Fig. 1C-1D and Fig. 2D-2F as new Fig 1 and reorganized initial Fig. 1A-1B and Fig. 2A-2C as new S1 Fig in revised manuscript. Since the genetic background and culture conditions of strains in new Fig. 1 are different from those in S1 Fig, and by presenting the whole zymogram we aims to highlight that the major catalase species regulated by *bub3^KO^* strains is *cat-3* but not the others. So we decided to keep these data as supporting information.

Reviewer #2:

This MS reports a novel role for proteins Bub3 and BuGZ in the transcriptional repression of the *catalase* gene *cat-3* in the fungus *Neurospora crassa*. In other eukaryotes Bub3 and its chaperone BuGZ have been involved in the spindle assembly checkpoint (SAC), a cell-cycle checkpoint that prevents the separation of the duplicated chromosomes until each chromosome is attached to the spindle. Therefore, the involvement of these proteins in the transcriptional regulation of a protein involved in ROS homeostasis is a novel and interesting finding. However, the authors do not clearly address weather this novel function is additional to their mitotic functions or simply these proteins play a different function (transcriptional regulation) in filamentous fungi. This should be made clear considering that the Author Summary states, “Our results not only expand the mechanism of catalase transcriptional regulation for maintaining redox homeostasis, but also imply that the cell cycle of fungi may be inextricably linked to its antioxidant processes”. This important claim can only be sustained if Bub3 and BuGZ also play a cell cycle role in *N. crassa*. Overall, the MS and the results are very clear. However, the authors need to address the following points:

Results

1. Line 124, Figure 1 and Figure 1 legend. WT strains are not labelled as bd or not bd in Figure 1. Colony diameter should be used in Figures 1B, D and similar figures, as actual growth rate was not determined in these experiments. Can the authors discuss why “the rescue phenotype was only seen when the *bub3^KO^* was in the *nonband* (*nbd*) background which has a wild-type *ras-1* gene [50]. Thus, subsequent experiments related to *bub3^KO^* transformants were carried out in this genetic background”?

Response: Special thanks for reviewer’s good comments. Due to the limitations of conditions, we really cannot fully prove that Bub3 and BuGZ also play a cell cycle role in *N. crassa*. So we have revised the relevant statement and hope to meet the reviewer’s requirements.

To follow the reviewer’s suggestion, we have labelled the genetic background (*bd* or *nbd*) in the revised manuscript.

As for the colony diameter, we measured that of all the strains when the hyphae of WT strain extend to the edge of the petri dish under 0 mM H_2_O_2_ treatment for the raw data. However, we need to quantitatively describe the H_2_O_2_ resistance of different strains. So we used the colony diameter of each strain under 0 mM H_2_O_2_ as a reference, dividing the colony diameter under treatment of other concentrations by the 0 mM-colony diameter to obtain the relative growth rate of this strain. Then the H_2_O_2_ resistance could be judged by comparing the relative growth rate between other strains and WT strains. Therefore, to emphasize the H_2_O_2_ resistance of strains, the actual growth rate didn’t show in quantitative figures. I hope our interpretation would satisfy the reviewer.

*band* (*bd*) mutation is the T79I point mutation in *ras-1* gene, which results in only 1/3 growth rate and more conidiation of wild-type strain (*nonband* strain). *ras-1^bd^* strain is an important tool as WT strain in the study of circadian rhythms for many years [1]. Since the regulation of circadian clock is another major direction of our laboratory, we have many strains with *bd* background for manipulation as well as the authentic wild-type *nbd* strains. When we screened the strains for H_2_O_2_ resistance, we found that single gene deletion of *bub3* or *bugz* under different genetic background (*bd* or *nbd*) were both H_2_O_2_ resistant. However, the genetic complementation experiments of *bub3^KO^* strains could only be successful under *nbd* background. Considering that the small membrane-anchored G-protein RAS is a central component in numerous signaling cascades that link extracellular stimuli to gene expression, we speculate that the *ras-1^bd^* mutation may prevent complemented Bub3 protein from playing its original function by interfering some signaling pathways.

2. Lines 146-148. Increased CAT-3 activity in *bub3^KO^* (*nbd*) mutant correlates with increased H_2_O_2_-resistance but does not confirm (prove) that H_2_O_2_-resistant phenotype observed in the mutant was “predominantly due to elevated CAT-3 activity”. There are other enzymes important for H_2_O_2_ detoxification such as the peroxiredoxins. It is therefore essential to perform the experiments in Figure 1, using a *bub3^KO^* (*nbd*) mutant lacking the *cat-3* gene. The results could drastically change some of the conclusions of this MS.

Response: Following the reviewer's advice, we not only genetically constructed the *bub3^KO^cat-3^KO^* and *bugz^KO^cat-3^KO^* double mutants and examined their H_2_O_2_ sensitivity (S3 Fig), but also investigated the effects of Bub3 and BuGZ on expression of two peroxiredoxin genes (*hyr1* and *prx1*) in *N. crassa* (S7A Fig). For a detailed description, please see the response to the second key question above.

3. Lines 186-191. The lack of Bub3 led to a considerable reduction of BuGZ protein levels and a *bugz^KO^* *bub3^KO^* double mutant show the same effects on CAT-3 expression. This suggests that *bub3^KO^* phenotype is due to BuGZ decreased levels. Yet, the phenotypes of the null mutants are so different in terms of growth and morphology. The authors need to discuss this. Is it possible that Bub3 and BuGZ do not work on the spindle assembly checkpoint (SAC) pathway in *N. crassa*?

Response: Considering the reviewer’s suggestion, we found that the growth and morphology of double mutant were identical to that of *bugz^KO^* strains by phenotypic examination (as shown below), confirming that the regulation of Bub3 for the growth of strain indeed depends on the BuGZ level. That is probably because BuGZ has a more significant function of regulating the global development of strains than Bub3 in *N. crassa*. Considering that the BuGZ protein in *bub3^KO^* strains does not drop to the level of *bugz^KO^* strains, we guess that the phenotypic differences between the single mutants may result from the small differences in BuGZ levels.

Due to the lack of evidence that Bub3 and BuGZ are involved in the SAC pathway in *N. crassa*, we cannot confirm whether they play roles in this process.

4. Lines 255-257 and Fig. 7G. The reduced enrichment of Myc-BuGZ with mutations of the zinc finger domains at the *cat-3* promoter are very minor compared to the wild-type BuGZ, while the effects on CAT-3 activity are very clear. Any comment on this?

Response: Considering the reviewer’s comments, we have revised the conclusions based on our ChIP data that the zinc fingers of BuGZ play other more important roles besides regulating its DNA binding to achieve *cat-3* repression. For a detailed explanation, please refer to the response to the first key question above.

Discussion

5. The authors state: “In agreement with reports that elevated ROS levels promote conidiation observed in the *cat-3^KO^* and *ras-1^bd^* strains [20, 50], we observed deficiencies in mycelial growth rates, hyphal formation, and conidial development in the *bub3^KO^* and *bugz^KO^* strains corresponding to the low levels of ROS caused by elevated CAT-3 expression”. However, ROS levels were not actually measured. More importantly, to prove this important point is necessary to perform the experiments using *bub3^KO^* and *bugz^KO^* mutants lacking the *cat-3* gene. In addition, the differences in mycelial growth rates, hyphal formation, and conidial development between *bub3^KO^* and *bugz^KO^* mutants were not addressed in this MS (for example no comments were made on Fig. 3A), and conidiation levels were not determined.

Response: To follow the reviewer’s suggestion, we constructed the *bub3^KO^cat-3^KO^* and *bugz^KO^cat-3^KO^* double mutants and found that there was no significant difference in growth phenotypes of *bub3^KO^* or *bugz^KO^* strains regardless of the presence or absence of the *cat-3* gene (S3 Fig), suggesting that our speculation in Disscussion is not valid. We have removed the relevant content according to the reviewer’s comments.

The different phenotype between *bub3^KO^* and *bugz^KO^* mutants was addressed above referring to response to point 3.

Minor

Peroxiredoxins are not mentioned in lines 65-67.

References on ROS signaling (1 and 4-6) are too old (outdated).

Reference 40 cited as evidence of “Bub3 and BubGZ being implicated in the occurrence of cancer” seems contradictory with the title and content of the reference: “Germline mutations in the spindle assembly checkpoint genes BUB1 and BUB3 are infrequent in familial colorectal cancer and polyposis”.

Line 264. “As our data”.

Response: Thanks for reviewer’s advice. We have carefully revised the manuscript according to your suggestions.

Reviewer #3:

The manuscript by He and colleagues has identified a novel function of the spindle assembly checkpoint protein Bub3 and its chaperone BuGZ in maintaining the redox homeostasis. The authors screened for mutations that confer resistance to ROS stress and surprisingly identified a mutation of the spindle checkpoint protein Bub3. They further demonstrated that Bub3, as well as its chaperone BuGZ, repress the expression of a catalase gene *cat-3*, and that Bub3 is required for BuGZ protein stability. With different mutations of BuGZ, they showed that BuGZ protein levels instead of its interaction with Bub3, regulates *cat-3* expression. In addition, they showed that BuGZ is localized at the *cat-3* locus and that the Zinc Finger domains of BuGZ are required for its recruitment. Finally, they showed that mutations of BuGZ lead to increased recruitment of transcription activators and the general transcription machinery to the *cat-3* gene.

Overall, the experiments are well-designed, the data are of high technical quality, and the conclusions are justified. The findings that spindle checkpoint protein regulates redox homeostasis is very novel and of broad interest. The manuscript is also well-written for a general audience. I only have some minor comments.

1. Line 136, the author mentioned nonband (*nbd*) background. It would be helpful to discuss what might be the impact of this background. I suppose that the different WT used in Figure 1A and 1C reflects background differences?

Response: Special thanks to the reviewer for your high recognition and good comments of this manuscript. According to the reviewer’s suggestion, we have labelled the genetic background of different WT used in Figure 1 as well as rearranged S1 Fig. For the discussion of impact of *nbd* and *bd* background, please refer to the response to Rev2 (point 1).

2. Fig6B, ChIP analysis shows that BuGZ is enriched at the *cat-3* locus. It would be interesting to see whether BuGZ binds to a control locus not involved in redox metabolism.

Response: As the reviewer’s advice, we have examined two negative control regions and also a positive control region for BuGZ ChIP binding (Fig. 5B). For a detailed description, please see the response to the first key question above.

3. Line 365, the authors state “…that the zinc finger domain of BuGZ is crucial for DNA binding…”. This conclusion needs to be tuned down as there is no DNA binding data of the zinc finger. ChIP analysis of BuGZ indicates its binding to chromatin is not sequence-specific, so zinc finger might have some other functions.

Response: Considering the reviewer's suggestion, we detected the specific recruitment of BuGZ to the *cat-3* region under both normal physiological conditions and oxidative stimulation, confirming the DNA binding specificity of BuGZ powerfully. For the detailed information, please refer to the response to the first key question above. However, it is true that the zinc fingers are almost useless for DNA binding. So we have revised the conclusions that the zinc fingers of BuGZ play other more important roles besides regulating its DNA binding to achieve *cat-3* repression.

**Reference**

1.	Belden WJ, Larrondo LF, Froehlich AC, Shi M, Chen CH, Loros JJ, et al. The band mutation in *Neurospora crassa* is a dominant allele of *ras-1* implicating RAS signaling in circadian output. Genes Dev. 2007;21(12):1494-1505.
