# Supplementary material for: A role for the mitotic proteins Bub3 and BuGZ in transcriptional regulation of catalase-3 expression
Source: PLoS Genet. 2022 Jun 6;18(6):e1010254. doi: 10.1371/journal.pgen.1010254 (PMC9203020; doi:10.1371/journal.pgen.1010254)
Supplement: Peer review history

# Peer review: Decision Letter 0

**DOI:** 10.1371/journal.pgen.1010254.r001

**Authors:** Aaron P. Mitchell, Gregory P. Copenhaver

27 Jan 2022

Dear Dr He,

Thank you very much for submitting your Research Article entitled 'A role for the mitotic proteins Bub3 and BuGZ in transcriptional regulation of catalase-3 expression' to PLOS Genetics.

The manuscript was fully evaluated at the editorial level and by independent peer reviewers. The reviewers appreciated the attention to an important problem, but raised some substantial concerns about the current manuscript. Based on the reviews, we will not be able to accept this version of the manuscript, but we would be willing to review a much-revised version. We cannot, of course, promise publication at that time.

Should you decide to revise the manuscript for further consideration here, your revisions should address the specific points made by each reviewer. We will also require a detailed list of your responses to the review comments and a description of the changes you have made in the manuscript.

If you decide to revise the manuscript for further consideration at PLOS Genetics, please aim to resubmit within the next 60 days, unless it will take extra time to address the concerns of the reviewers, in which case we would appreciate an expected resubmission date by email to plosgenetics@plos.org.

If present, accompanying reviewer attachments are included with this email; please notify the journal office if any appear to be missing. They will also be available for download from the link below. You can use this link to log into the system when you are ready to submit a revised version, having first consulted our Submission Checklist.

To enhance the reproducibility of your results, we recommend that you deposit your laboratory protocols in protocols.io, where a protocol can be assigned its own identifier (DOI) such that it can be cited independently in the future. Additionally, PLOS ONE offers an option to publish peer-reviewed clinical study protocols. Read more information on sharing protocols at https://plos.org/protocols?utm_medium=editorial-email&utm_source=authorletters&utm_campaign=protocols

Please be aware that our data availability policy requires that all numerical data underlying graphs or summary statistics are included with the submission, and you will need to provide this upon resubmission if not already present. In addition, we do not permit the inclusion of phrases such as "data not shown" or "unpublished results" in manuscripts. All points should be backed up by data provided with the submission.

While revising your submission, please upload your figure files to the Preflight Analysis and Conversion Engine (PACE) digital diagnostic tool.  PACE helps ensure that figures meet PLOS requirements. To use PACE, you must first register as a user. Then, login and navigate to the UPLOAD tab, where you will find detailed instructions on how to use the tool. If you encounter any issues or have any questions when using PACE, please email us at figures@plos.org.

PLOS has incorporated Similarity Check, powered by iThenticate, into its journal-wide submission system in order to screen submitted content for originality before publication. Each PLOS journal undertakes screening on a proportion of submitted articles. You will be contacted if needed following the screening process.

To resubmit, use the link below and 'Revise Submission' in the 'Submissions Needing Revision' folder.

[LINK]

We are sorry that we cannot be more positive about your manuscript at this stage. Please do not hesitate to contact us if you have any concerns or questions.

Yours sincerely,

Aaron P. Mitchell, PhD

Associate Editor

PLOS Genetics

Gregory P. Copenhaver

Editor-in-Chief

PLOS Genetics

The reviewers appreciated the novelty of the findings, as did I. However, the two key questions that need to be addressed are:

1. How does BUGZ bind to or associate with DNA? You will see that Rev2 (point 4) and Rev3 (point 3) both mention his issue, and I reviewed Figure 7 carefully as well. The impact of the ZNF mutations on ChIP outcome seems marginal, especially when compared with a BUGZ deletion mutant in Figure 6. I share the reviewers' concern that the DNA interaction is nonspecific. You need to examine a control gene regulatory regions for ChIP binding. These would be genes whose expression is unaffected by a BUGZ deletion. Note that simply toning down the conclusion as recommended by Rev 3 would not make the manuscript acceptable at PLOS Genetics, because the level of the advance would not be sufficient.

2. Rev2 (point 2) mentions a concern that I share, which is that cat-3 may not be the sole relevant target of BUGZ. Please follow the reviewers' suggestion or test expression of other relevant genes, such as those encoding peroxiredoxins.

Please note as well that the promoter region used for ChIP assays in Figure 7 is not indicated in the legend, unlike the situation with Figure 6. Please attend to that.

Reviewer's Responses to Questions

**Comments to the Authors:**

**Please note here if the review is uploaded as an attachment.**

Reviewer #1: In this manuscript, the authors unraveled a novel function for proteins better known as involved in spindle assembly, namely transcription regulation, as well as providing details of the likely underlying mechanism. They investigated the role of the spindle assembly checkpoint proteins Bub3 and BuGZ in the transcriptional regulation of the cat-3 gene in the filamentous fungus Neurospora crassa. They found that the proteins negatively regulate cat-3 transcription, depending particularly on BuGZ levels and its DNA binding and not on BuGZ/Bub3 interactions. This DNA binding hampers the recruitment of transcription activators required for transcription. The authors also compare the situation in different organisms. The manuscript is well and concisely written and nice to read.

Minor point: I wonder if Figs. 1A and 1B are really required, because the same information is shown in Figs. 1C and 1D, respectively. The same applies to Figs. 2A, 2B and 2C, with the information repeated in Figs. 2C, 2D and 2F, respectively.

Reviewer #2: Uploaded as an attachment

Reviewer #3: The manuscript by He and colleagues has identified a novel function of the spindle assembly checkpoint protein Bub3 and its chaperone BuGZ in maintaining the redox homeostasis. The authors screened for mutations that confer resistance to ROS stress and surprisingly identified a mutation of the spindle checkpoint protein Bub3. They further demonstrated that Bub3, as well as its chaperone BuGZ, repress the expression of a catalase gene cat-3, and that Bub3 is required for BuGZ protein stability. With different mutations of BuGZ, they showed that BuGZ protein levels, instead of its interaction with Bub3, regulates cat-3 expression. In addition, they showed that BuGZ is localized at the cat-3 locus and that the Zinc Finger domains of BuGZ are required for its recruitment. Finally, they showed that mutations of BuGZ lead to increased recruitment of transcription activators and the general transcription machinery to the cat-3 gene.

Overall, the experiments are well-designed, the data are of high technical quality, and the conclusions are justified. The findings that spindle checkpoint protein regulates redox homeostasis is very novel and of broad interest. The manuscript is also well-written for a general audience. I only have some minor comments.

1. Line 136, the author mentioned nonband (nbd) background. It would be helpful to discuss what might be the impact of this background. I suppose that the different WT used in Figure 1A and 1C reflects background differences?

2. Fig6B, ChIP analysis shows that BuGZ is enriched at the cat-3 locus. It would be interesting to see whether BuGZ binds to a control locus not involved in redox metabolism.

3. Line 365, the authors state “…that the zinc finger domain of BuGZ is crucial for DNA binding…”. This conclusion needs to be tuned down as there is no DNA binding data of the zinc finger. ChIP analysis of BuGZ indicates its binding to chromatin is not sequence-specific, so zinc finger might have some other functions.

**********

**Have all data underlying the figures and results presented in the manuscript been provided?**

Large-scale datasets should be made available via a public repository as described in the *PLOS Genetics* data availability policy, and numerical data that underlies graphs or summary statistics should be provided in spreadsheet form as supporting information.

Reviewer #1: Yes

Reviewer #2: Yes

Reviewer #3: Yes

**********

PLOS authors have the option to publish the peer review history of their article (what does this mean?). If published, this will include your full peer review and any attached files.

If you choose “no”, your identity will remain anonymous but your review may still be made public.

**Do you want your identity to be public for this peer review?** For information about this choice, including consent withdrawal, please see our Privacy Policy.

Reviewer #1: No

Reviewer #2: No

Reviewer #3: **Yes: **Songtao Jia

Attachment Submitted filename: Cat-3 PLoS Genetics Review 2022.docx Click here for additional data file.

# Author comment: Author response to Decision Letter 0

**DOI:** 10.1371/journal.pgen.1010254.r002

29 Apr 2022

Attachment Submitted filename: Response to reviewers.docx Click here for additional data file.

# Peer review: Decision Letter 1

**DOI:** 10.1371/journal.pgen.1010254.r003

**Authors:** Aaron P. Mitchell, Gregory P. Copenhaver

13 May 2022

Dear Dr He,

We are pleased to inform you that your manuscript entitled "A role for the mitotic proteins Bub3 and BuGZ in transcriptional regulation of catalase-3 expression" has been editorially accepted for publication in PLOS Genetics. Congratulations!

Before your submission can be formally accepted and sent to production you will need to complete our formatting changes, which you will receive in a follow up email. Please be aware that it may take several days for you to receive this email; during this time no action is required by you. Please note: the accept date on your published article will reflect the date of this provisional acceptance, but your manuscript will not be scheduled for publication until the required changes have been made.

Once your paper is formally accepted, an uncorrected proof of your manuscript will be published online ahead of the final version, unless you’ve already opted out via the online submission form. If, for any reason, you do not want an earlier version of your manuscript published online or are unsure if you have already indicated as such, please let the journal staff know immediately at plosgenetics@plos.org.

In the meantime, please log into Editorial Manager at https://www.editorialmanager.com/pgenetics/, click the "Update My Information" link at the top of the page, and update your user information to ensure an efficient production and billing process. Note that PLOS requires an ORCID iD for all corresponding authors. Therefore, please ensure that you have an ORCID iD and that it is validated in Editorial Manager. To do this, go to ‘Update my Information’ (in the upper left-hand corner of the main menu), and click on the Fetch/Validate link next to the ORCID field.  This will take you to the ORCID site and allow you to create a new iD or authenticate a pre-existing iD in Editorial Manager.

If you have a press-related query, or would like to know about making your underlying data available (as you will be aware, this is required for publication), please see the end of this email. If your institution or institutions have a press office, please notify them about your upcoming article at this point, to enable them to help maximise its impact. Inform journal staff as soon as possible if you are preparing a press release for your article and need a publication date.

Thank you again for supporting open-access publishing; we are looking forward to publishing your work in PLOS Genetics!

Yours sincerely,

Aaron P. Mitchell, PhD

Associate Editor

PLOS Genetics

Gregory P. Copenhaver

Editor-in-Chief

PLOS Genetics

www.plosgenetics.org

Twitter: @PLOSGenetics

----------------------------------------------------

Comments from the reviewers (if applicable):

Reviewer's Responses to Questions

**Comments to the Authors:**

**Please note here if the review is uploaded as an attachment.**

Reviewer #1: The authors have deal well with the comments and suggestions made by the reviewers and changed the manuscript accordingly.

Reviewer #2: In this revised version of this MS, the authors have addressed all the questions and comments I made to the original version.

Reviewer #3: The revised manuscript has satisfactorily addressed my concerns. The additional data strengthen the manuscript and the revisions in text are more accurate and thorough. I recommend acceptance for publication.

**********

**Have all data underlying the figures and results presented in the manuscript been provided?**

Large-scale datasets should be made available via a public repository as described in the *PLOS Genetics* data availability policy, and numerical data that underlies graphs or summary statistics should be provided in spreadsheet form as supporting information.

Reviewer #1: Yes

Reviewer #2: Yes

Reviewer #3: Yes

**********

PLOS authors have the option to publish the peer review history of their article (what does this mean?). If published, this will include your full peer review and any attached files.

If you choose “no”, your identity will remain anonymous but your review may still be made public.

**Do you want your identity to be public for this peer review?** For information about this choice, including consent withdrawal, please see our Privacy Policy.

Reviewer #1: No

Reviewer #2: **Yes: **Jesús Aguirre

Reviewer #3: No

----------------------------------------------------

**Data Deposition**

If you have submitted a Research Article or Front Matter that has associated data that are not suitable for deposition in a subject-specific public repository (such as GenBank or ArrayExpress), one way to make that data available is to deposit it in the Dryad Digital Repository. As you may recall, we ask all authors to agree to make data available; this is one way to achieve that. A full list of recommended repositories can be found on our website.

The following link will take you to the Dryad record for your article, so you won't have to re‐enter its bibliographic information, and can upload your files directly: 

http://datadryad.org/submit?journalID=pgenetics&manu=PGENETICS-D-21-01690R1

More information about depositing data in Dryad is available at http://www.datadryad.org/depositing. If you experience any difficulties in submitting your data, please contact help@datadryad.org for support.

Additionally, please be aware that our data availability policy requires that all numerical data underlying display items are included with the submission, and you will need to provide this before we can formally accept your manuscript, if not already present.

----------------------------------------------------

**Press Queries**

If you or your institution will be preparing press materials for this manuscript, or if you need to know your paper's publication date for media purposes, please inform the journal staff as soon as possible so that your submission can be scheduled accordingly. Your manuscript will remain under a strict press embargo until the publication date and time. This means an early version of your manuscript will not be published ahead of your final version. PLOS Genetics may also choose to issue a press release for your article. If there's anything the journal should know or you'd like more information, please get in touch via plosgenetics@plos.org.

# Editor report: Acceptance letter

**DOI:** 10.1371/journal.pgen.1010254.r004

**Authors:** Aaron P. Mitchell, Gregory P. Copenhaver

1 Jun 2022

PGENETICS-D-21-01690R1 

A role for the mitotic proteins Bub3 and BuGZ in transcriptional regulation of *catalase-3* expression 

Dear Dr He, 

We are pleased to inform you that your manuscript entitled "A role for the mitotic proteins Bub3 and BuGZ in transcriptional regulation of *catalase-3* expression" has been formally accepted for publication in PLOS Genetics! Your manuscript is now with our production department and you will be notified of the publication date in due course.

The corresponding author will soon be receiving a typeset proof for review, to ensure errors have not been introduced during production. Please review the PDF proof of your manuscript carefully, as this is the last chance to correct any errors. Please note that major changes, or those which affect the scientific understanding of the work, will likely cause delays to the publication date of your manuscript. 

Soon after your final files are uploaded, unless you have opted out or your manuscript is a front-matter piece, the early version of your manuscript will be published online. The date of the early version will be your article's publication date. The final article will be published to the same URL, and all versions of the paper will be accessible to readers.

Thank you again for supporting PLOS Genetics and open-access publishing. We are looking forward to publishing your work! 

With kind regards,

Agnes Pap

PLOS Genetics

On behalf of:

The PLOS Genetics Team

Carlyle House, Carlyle Road, Cambridge CB4 3DN | United Kingdom

plosgenetics@plos.org | +44 (0) 1223-442823

plosgenetics.org | Twitter: @PLOSGenetics
